# Raman and Infrared Spectroscopy of Materials for Lithium-Ion Batteries

**DOI:** 10.3390/ijms262411879

**Published:** 2025-12-09

**Authors:** Christian M. Julien, Alain Mauger

**Affiliations:** Institut de Minéralogie, Physique des Matériaux et de Cosmologie (IMPMC), Sorbonne Université, UMR-CNRS 7590, 4 place Jussieu, 75252 Paris, France; alain.mauger@sorbonne-universite.fr

**Keywords:** vibrational spectroscopy, Raman and FTIR spectroscopy, Li-ion batteries, cathodes, anodes, electrolytes

## Abstract

Vibrational spectroscopy is one of the most powerful techniques available for the characterization of materials for Li-ion batteries (LIBs) and one of the most useful tools when X-ray diffraction is ineffective for amorphous substances. Raman spectroscopy is essentially a probe to examine the surface of compounds that strongly absorb visible light, which is the case for all electrode materials, while infrared spectroscopy is a tool that examines the entire volume of particles. The purpose of this review is to study the lattice dynamics of cathode, anode, and electrolyte materials of advanced LIBs, especially nanomaterials for high-power-density application. Ex situ and in situ analyses are presented, which satisfy several key issues, such as structural stability over long-term cycling.

## 1. Introduction

The characterization of the materials building lithium-ion batteries (electrodes and electrolytes) and the ultimate goal of correlating structural characteristics with physical and chemical properties require the use of a broad range of techniques. Among them, vibrational spectroscopy is one of the most powerful [1]. Raman scattering (RS) and Fourier transform infrared (FTIR) spectroscopies are local probes of structural properties that replace X-ray diffraction, which is ineffective for analyzing highly disordered or amorphous substances. The vibrational patterns of a molecule investigated by infrared absorption and Raman scattering results from the forces arising from the interactions between electrons and nuclei. These spectroscopies allow access to the energy of the different fundamental vibrational modes, which are the spectral fingerprints of the molecules or complex ions studied. Thus, the values of the force constants of oscillators are collected from the frequencies of the observed modes and can hopefully be correlated with the electronic structure using bonding theories. As a first approximation, spectra result from the superposition of the components of all local entities present in similar materials, unlike diffraction data, which provide a weighted average of similar interplanar spacings. Generally, the frequencies and relative intensities of the spectral features are sensitive to coordination geometry and oxidation states. Thus, the morphology, grain size, or degree of long-range order of the crystal lattice do not strongly affect the appearance of the spectra.

However, it is important to mention that Raman spectroscopy is essentially a probe for investigating the surface of solids that strongly absorb visible light. This is the case for all electrode materials utilized in Li-ion batteries, i.e., carbons, silicon, transition-metal oxides, polyphosphates, etc. In contrast, infrared spectroscopy is an analytical tool that examines the entire volume of compounds. It is also relevant to point out that, in terms of selection rules, RS and FTIR are complementary techniques, since Raman spectroscopy studies phonons at the center of Brillouin (*q* = 0), whereas FTIR spectroscopy investigates the entire Brillouin zone (0 < *q* < 2/*a*), where *q* is the wave vector, and *a* is the lattice parameter. One sensitive case is the lattice dynamics of the layered transition-metal oxides LiMO_2_ (*M* = Ni, Co, Mn), which reveals the lithium-cage mode allowed in infrared. Therefore, FTIR allows probing of the local environment of lithium in the Li_x_*M*O_2_ electrode during charge–discharge cycles.

The purpose of this review is to present some typical examples of Raman and FTIR spectroscopic investigations of materials building lithium-ion batteries (LIBs). Negative (anode) and positive (cathode) electrodes and liquid and solid-state electrolytes (SSEs) with various chemistries are presented. This chapter is organized as follows. Spectroscopy background, including vibrations of nanoparticles and Raman resonance and Raman mapping experiments, are presented in Section 2. Tools used for the analysis of spectra, i.e., band deconvolution, and polarization analyses are examined in Section 3. Section 4 reports vibrational features of cathode materials, including lamellar, spinel, and olivine structure. The Raman and FTIR spectra of the various anodes, i.e., carbonaceous and silicon nanoparticles and titanates, are presented in Section 5. Finally, Section 6 is devoted to the intrinsic vibrational modes of liquid and solid-state electrolytes, including crystalline and glassy phases of oxide- and sulfide-based superionic compounds. Spectroscopy patterns of the solid electrolyte interphase are also considered.

## 2. Spectroscopy Background

### 2.1. Photon–Matter Interactions

**Figure 1:**
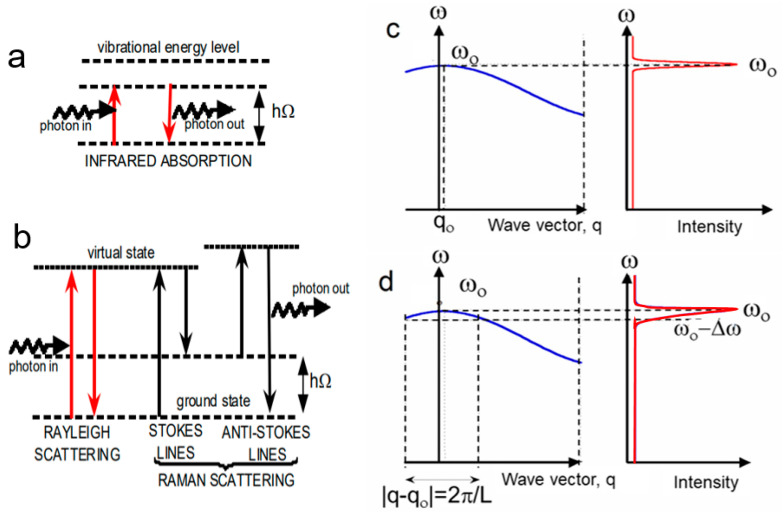
Energy level diagram for different processes, including transitions involved in (**a**) infrared absorption and (**b**) Raman and Rayleigh scattering. The molecular vibration in the sample is of a frequency ω. Dispersion curves (blue lines) and Raman responses (red lines) for (**c**) single crystal and (**d**) nanoparticle.

Classically, light–matter interactions are a result of an oscillating electromagnetic field resonantly interacting with charged particles. The interactions that light can have with matter include emission, absorption, transmission, reflection, and scattering. The Raman scattering effect is a change in the wavelength of light that occurs when a light beam is deflected by molecules. Figure 1a,b represents the energy level diagram for different light–matter processes. Infrared absorption takes place when the energy of the incident photon *h*ν_i_ is equal to the energy difference *h*Ω between two states of the molecule (Figure 1a), where *h* is the Planck constant. Unabsorbed photons are scattered, and incident photons do not need to be in resonance with two states of a molecule for the scattering to occur. Rayleigh scattering (photon elastically scattered) corresponds to the process without any change in the atomic coordinates of the molecule; thus, ν_scatt_ = ν_i_. The process leading to an inelastic scattering constitutes the Raman effect, such that the energies of the incident and scattered photons are no longer equal. The Stokes Raman scattering is the transfer of energy in a virtual state from the photon to the molecule, such as ν_S_ = ν_i_ − Ω, while the anti-Stokes Raman scattering refers to the transfer of energy in the virtual state from the molecule to the photon, such as ν_AS_ = ν_i_ + Ω (Figure 1b).

Raman scattering occurs during the interaction between an incident photon and the electric dipole of a molecule. Although the Raman spectrum displays vibrational frequencies, it can be considered an electronic (more precisely, vibronic) spectroscopy. The dipole moment $\vec{P}$ of a molecule is proportional to the external electric field $\vec{E}$ applied as follows: $\vec{P}$ = α$\vec{E}$, where α represents the polarizability of the molecular tensor. For an incident radiation of frequency ν_i_, the electric field is a time-varying quantity of the form *E*_i_ = *E*_o_ cos(2πν_i_t). In classical terms, the interaction can be viewed as a perturbation of the polarizability as follows:

(1) $$=\alpha_{o}+\sum_{k}\left(\frac{\partial \alpha }{\partial X_{k}}\right)\Delta X_{k}+higher\;terms.$$

The polarizability of a vibrating molecule is also a time-varying quantity that is a function of its vibronic frequency, Ω, expressed by α = α_0_ + α_m_ cos(2πΩt). Therefore, the polarizability of the oscillating dipole is expressed as follows:

(2) $$P=\alpha_{o}E_{o}\cos(2\pi \nu_{i}t)+\alpha_{m}E_{o}\frac{\partial \alpha }{\partial X_{k}}\cos(2\pi \nu_{i}t)\cos(2\pi \Omega t),$$

(3) $$P=\alpha_{o}E_{o}\cos(2\pi \nu_{i}t)+\frac{\alpha_{m}E_{o}}{2}\frac{\partial \alpha }{\partial X_{k}}\left[\cos2\pi \left(\nu_{i}-\Omega \right)t+\cos2\pi (\nu_{i}+\Omega )t)\right].$$

In Equation (3), the dipole moment is a function of three component frequencies, ν_i_, (ν_i_ − Ω), and (ν_i_ + Ω), which, respectively, generate the Rayleigh scattering, Stokes, and anti-Stokes frequencies, as shown in the energy diagram in Figure 1. The frequency shift between the incident radiation frequency, ν_i_, and those of the Raman anti-Stokes and Stokes lines corresponds to the vibronic frequencies Ω_i_ of the molecule. The intensity ratio of the anti-Stokes and Stokes lines is given by the following [2]:

(4) $$\frac{I_{A}}{I_{S}}={\left(\frac{\nu_{i}+\Omega }{\nu_{i}-\Omega }\right)}^{4}\exp\left(\frac{-h\Omega }{k_{B}T}\right).$$

### 2.2. Site Symmetry and Vibrational Modes

Crystallographic data recording for alkali-containing oxides shows that each alkali cation presents a preferred coordination geometry, including cases of the cation site occupancies with different coordination numbers (*CN*) in the same crystal. For example, in the spinel structure (space group *Fd3m*), Li^+^ ions exhibit a strong tendency towards four-fold coordination with oxygen atoms, whereas the coordination number of Li^+^ ions is six-fold in the layered lattice (space group *R*$\bar{3}$*m*). The coordination numbers of alkali cations in the powdered oxide state are similar to those observed in crystals. For simplicity, it is assumed that the alkali cations occupy sites in the crystal lattice with different coordination numbers (*CN* = 4, 6, 8) and that these sites have ideal tetrahedral (*T_d_*), octahedral (*O_h_*), and cubic (*O_h_*) configurations, respectively. Group theory then predicts the following irreducible representations for the *M*-O (site) stretching modes in the case of tetrahedral (*Γ_tet_*), octahedral (*Γ_oct_*), and cubic (*Γ_cub_*) configuration, in standard notation:

*Γ_tet_* = *A*_1_ (R) + *F*_2_ (IR, R), (5)

*Γ_oct_* = *A*_1*g*_ (R) + *E_g_* (R) + *F*_1*u*_ (IR, R), (6)

*Γ_cub_* = *A*_1*g*_ (R) + *A*_2*u*_ (ia) + *F*_2*g*_ (R) + *F_u_* (IR, R), (7)

where (IR), (R), and (ia) denote infrared-active, Raman-active, and inactive modes, respectively. According to these equations, only one infrared-active mode exists in a triply degenerate state. Since the anionic sites are almost always irregular, site distortions lead to a reduction in symmetry and, consequently, a splitting of the *T* modes occurs. For instance, the *F*_2_ mode for a *T_d_* site would produce two infrared-active bands (*A*_1_, *E*) for a *C*_3*v*_ site symmetry, and would split into three infrared bands (*A*_1_, *B*_1_, *B*_2_) when the symmetry of the site is further reduced to *C*_2*v*_.

It is well-known that the cation–oxygen distance *r_M-O_* and the vibrational reduced mass µ depend on the coordination geometry. The values of ionic radii, tabulated by Shannon, allow the calculation of the cation–oxygen distance, *r_M-O_* = *r_M_* + *r_O_*, where *r_M_* is the radius of the cation and *r_O_* = 1.40 Å. The cation–oxygen internuclear distances for coordination numbers 4 ≤ *CN* ≤ 12, shown in Table 1, exhibit an increase in *r_M-O_* with the coordination number and match well with the values obtained from crystallography [3].

Let us now determine the reduced mass of the cation vibration. In general, the reduced mass is given by *μ* = *G*_ij_^−1^, where *G_ij_* is the appropriate element of the symmetry-factored G matrix in Wilson’s matrix approach [4]. G matrix elements have been determined by derivation for different point-group symmetries. In particular, the reduced mass for tetrahedral (*µ*_4_), octahedral (*µ*_6_), and cubic (*µ*_8_) symmetries are as follows:

(8) $$\mu_{4}=\frac{3m_{C}m_{A}}{3m_{C}+4m_{A}},$$

(9) $$\mu_{6}=\frac{m_{C}m_{A}}{m_{C}+2m_{A}},$$

(10) $$\mu_{8}=\frac{3m_{C}m_{A}}{3m_{C}+8m_{A}},$$

where *m_C_* and *m_A_* are the mass of the cation and oxygen, respectively. For example, Equation (9) has been employed by Exarhos et al. [5] to calculate the reduced mass for cation vibration in alkali-metal and alkaline-earth metaphosphate glasses. From the expression of the vibrational frequency of the symmetrical stretching mode, the vibrational force constant, *F*, is given by the following:

(11) $$F=4\pi^{2}c^{2}\mu \nu^{2},$$

where *c* is the speed of light. When recorded by infrared spectroscopy, the cation vibration frequencies and their compositional dependence can elucidate the role of the alkali-metal cation on the LiMO_2_ framework [6]. The dependence of the frequency of the cation displacement on the symmetry and site size of the anionic lattice was investigated using a simplified version of the Born–Mayer potential to describe cation–lattice interactions. Assuming that each alkali-metal cation is surrounded by six neighboring oxygen atoms arranged in an octahedral configuration, the following expression holds for the square of the cation displacement frequency:

(12) $$\nu^{2}=\left(\frac{\alpha }{48\pi^{2}c^{2}\varepsilon_{0}}\right)\frac{q_{C}q_{A}}{\mu r_{M-O}^{3}},$$

where *q_C_* and *q_A_* represent the charge of the cation and anionic site, respectively, *µ* is the vibrational reduced mass, and *r_M-O_* corresponds to the cation–oxygen equilibrium distance. *ε*_0_ represents the permittivity of the free space, and *α* is a pseudo-Madelung constant. The Madelung constant rests on the site symmetry (coordination number). With *CN* = 4, it is *α* = 1.638 for the zinc blende structure; it is *α* = 1.747 for the sodium chloride lattice with *CN* = 6; and with *CN* = 8, it is *α* = 1.762 for the CsCl structure. Finally, the relationship between the force constant *F* expressed in N cm^−1^ and the average *M*-O bond length *r*_M-O_ expressed in Å is given by the following:

(13) $$F=\frac{17}{r_{M-O}^{3}}.$$

The lithium transition-metal oxides (TMOs) used as cathode materials in rechargeable batteries display different crystallographic structures. Furthermore, their phase diagram during lithium insertion/extraction is rather complex. Three classes of materials are currently investigated: the spinel-type, the rock-salt-type, and polyanionic structures. Table 2 lists the Raman-active bands of these compounds as a function of the lattice symmetry. It is worth noting that the number of modes increases significantly for the tetragonal or ordered spinel frameworks.

### 2.3. Vibration of Nanoparticles

It is well-known that frequency shift in the Raman spectrum can arise from various effects. Tensile and compressive stresses affect the Raman line by shifting it towards the red or towards the blue, respectively. A downshift is also observed in the case of a sample made up of thin slabs or nanoparticles [7,8]. For a perfect single crystal of infinite size, the internal, external, and lattice modes (phonons) are represented by well-defined plane waves, characterized by a wave vector ***q***. Since the frequency of the excitation light *c* is much greater than the frequency of phonons, only phonons close to the center of the Brillouin zone are allowed in Raman spectroscopy (|***q***| = *ω*/*c* ≈ 0, where *ω* = 2πν) (Figure 1c). For a spherical nanoparticle of diameter *L*, the vibration is confined to the volume of the crystallite. Consequently, the phonon wave vector spectrum is broadened and exhibits a finite width on the order of 2π/*L* around the zone center (Figure 1d). This effect induces an asymmetric broadening and a frequency shift in the observed phonon by relaxing the selection rule *q* = 0. Single-phonon asymmetric Raman bands were observed for the first time in small particles. The Gaussian phonon confinement model was proposed by Richter et al. [9]. This model is briefly examined in the following. For a particle of diameter *L*, the Raman intensity *I*(*ω*, *L*) at frequency shift *ω*/2π relative to the excitation line is expressed by the following relation (14) [9]:

(14) $$I\left(\omega ,d\right)=I_{o}\int_{O}^{L}\frac{{C(q)}^{2}}{{\left[\omega -\omega_{o}(q,T)\right]}^{2}+{\left(\Gamma (T)/2\right)}^{2}}$$

For a spherical nanoparticle, the Gaussian weighting function (i.e., the phonon dispersion curve being isotropic) is *C*(0,q)^2^ = *C*_o_ exp[−½(*qL*/*a*)^2^], where *a* represents the lattice constant of the material. Equation (14) is shown as the sum of weighted Lorentzian components to the Raman band from each bulk phonon of wave vector ***q***. So far, only bulky vibrational modes are regarded, while surface modes can also contribute, especially when the particles are of nanometric size, with a larger surface/volume ratio. For example, vibrational Raman modes of the surface of nucleated cordierite glasses were observed at very low frequencies. They have been analyzed as a function of the size of spherical microcrystallites with spinel structure, and results show that the Raman band frequency is proportional to the inverse diameter of the particles [10].

### 2.4. Raman Single-Point and Mapping Measurements

#### 2.4.1. Experimental In Situ Raman Setups

**Figure 2:**
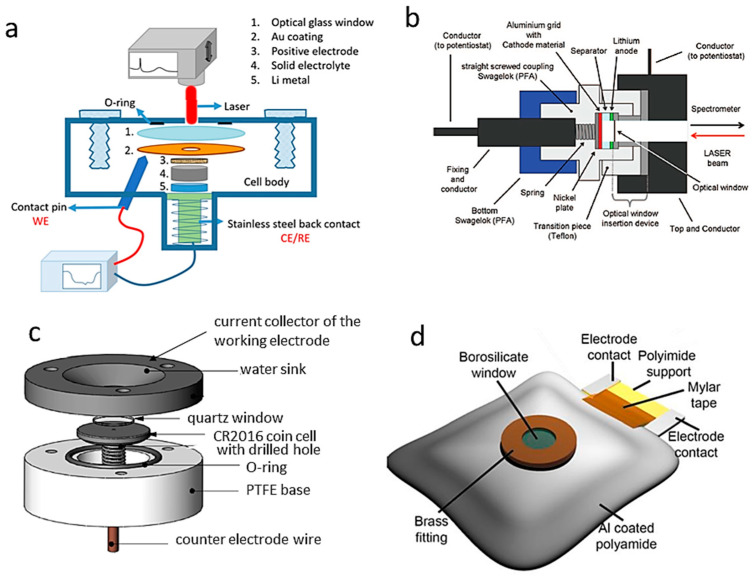
(**a**) Scheme of in situ Raman spectroelectrochemical cells. WE: working electrode, and CE/RE: counter electrode/reference electrode. Reproduced with permission from [13]. Copyright 2020 Wiley. (**b**) Swagelok-type cell with sapphire optical window. Reproduced with permission from [15]. Copyright 2013 Elsevier. (**c**) In situ setup with CR2016 coin cell adapted with quartz window. Reproduced with permission from [17]. Copyright 2017 Elsevier. (**d**) Pouch-cell with borosilicate window. Reproduced with permission from [18]. Copyright 2015 Wiley.

In situ Raman spectroscopy of materials for batteries is generally performed using a confocal Raman spectrometer equipped with a microscope with backscattering geometry. In situ Raman electrochemical cells are currently equipped with a window made of glass, quartz, or sapphire that is transparent to the excitation laser light. A long working distance optical objective with 100× magnification is used, producing a laser spot of approximately 3 μm^2^ on the sample electrode. For a description of the micro-Raman device, we refer the reader to the review articles in references [11,12]. Figure 2 shows examples of typical spectroelectrochemical cells for in situ Raman studies [13]. Gross and colleagues reported in situ measurements using a Swagelok-type cell with a sapphire optical window [14,15]. Burba and Frech described a spectroelectrochemical cell made from a modified industrial button cell with a 2 mm diameter hole drilled in its casing [16]. In situ Raman spectra were recorded on Li//V_2_O_5_ cells to identify the different phases present during electrode lithiation and to evaluate electrochemical performance during charge/discharge cycling. Another in situ cell was constructed using a standard Swagelok device equipped with a sapphire optical window (Figure 2b) [15]. Standard 2032 button cells (from Pred Materials International) were adapted to perform in situ Raman mapping of the electrode materials (Figure 2d). Fang et al. employed a MgO window to cover a 1/8-inch diameter drilled hole. State-of-charge mappings were recorded on a 35 × 35 µm^2^ area considering two spectral patterns, i.e., the position and intensity of the Raman peak [17]. Ghanty et al. [18] developed an in situ Raman pouch-cell incorporating a sodium-borosilicate optical window mounted on an aperture in the aluminum-coated polyamide casing. This design allows for real-time Raman spectra during the polarization of nickel-rich Li_1+x_(Ni_y_Co_z_Mn_z_)_w_O_2_ electrodes while remaining fully compatible with routine electrochemical tests. Combined in situ analysis by X-ray diffraction and Raman spectroscopy revealed the structural evolution of the lamellar material from the hexagonal H1 phase to the H2 phase, as well as the coexistence of the two phases in the biphase region at 4.2–4.3 V vs. Li^+^/Li.

#### 2.4.2. Optical Skin Depth

For Raman spectroscopy involving monochromatic light falling on the sample being analyzed, the response depends on the excited volume. Thus, the penetration depth depends on the transparency of the sample. Therefore, axial resolution is determined by the optical penetration depth (*δ*_p_) in the case of an opaque material. For example, due to the strong optical absorption of the visible light by electrode materials, the observed Raman spectra originate only from a thin surface layer. Due to its low *δ*_p_ value, Raman spectroscopy can, in most cases, be considered a surface analysis technique. The Raman band intensity is then low due to the small material volume analyzed (*V* < 1 µm^3^). The penetration depth is expressed by the following relationship:

*δ*_p_ = (2*λ*/*μσ*)^1/2^ (15)

where *λ* represents the excitation wavelength, *σ* is the electronic conductivity of the sample, and *μ* is its magnetic permeability. Therefore, using laser excitation with a longer wavelength increases the penetration depth, and an increase in electronic conductivity, for example, from the transition from semiconductor to metal, leads to a decrease in *δ*_p_. According to Beer–Lambert’s law, the intensity *I*(ξ) of laser light inside materials is an exponential function of the material’s absorption coefficient α: *I*(ξ) = *I*_0_ exp[−*αξ*]. Since the optical penetration depth is defined as the distance at which the intensity decreases to 1/*e* (37%), where *e* is the base of the logarithmic system, we have *δ*_p_ = α − 1. For example, *δ*_p_ ≈ 1 µm for crystalline silicon, *δ*_p_ ≈ 100 nm for amorphous silicon, and *δ*_p_ ≈ 100 nm for Li_x_Si [19]. Currently, Raman experiments are performed with an excitation line at very low power *W*_laser_ ≈ 0.02 mW using a 0.1% filter, corresponding to a specific power density of 600 W·cm^−2^. The optical penetration depth is less than 50 nm in highly oriented pyrolytic graphite, probed with the 514.5 nm green laser line [20], while *δ*_p_ reduces to few nanometers in Li_1−x_Ni_1/3_Mn_1/3_Co_1/3_O_2_ delithiated cathode material. Another example encountered in surface-modified materials is the shallow optical penetration depth (*δ*_p_ ≈ 30 nm) of carbon-coated LiFePO_4_ particles [21]. Therefore, to probe these electrodes, a laser excitation line with a high wavelength λ > 700 nm is preferred to the 532 nm excitation radiation of a solid-state laser.

#### 2.4.3. In Situ Raman Imaging

In situ Raman mapping has been developed to relate chemical information with spatial characterization at multiple points. This technique can also determine heterogeneity of particles during the cell’s charge–discharge process. It is powerful for studying electrode degradation. A Raman image is constructed by a set of hyperspectral data, where each pixel includes a well-defined Raman spectrum. Raman microspectroscopy is a spatially resolved technique, currently used to analyze electrodes in situ by generating local surface images, constructing state-of-charge (SOC) maps, or studying the kinetics of insertion/extraction reactions in a single particle. The feasibility of Raman mapping was first explored by Panitz et al. [22], who analyzed the lithium intercalation in a graphite electrode under potentiostatic and galvanostatic charge/discharge cycles. Their results indicated that there was a non-homogeneous lithium insertion in the graphite electrode at a potential of 0.2 V vs. Li^+^/Li, and that a new Raman band evolves at 1850 cm^−1^ at a potential of 0.18 V vs. Li^+^/Li. This band was tentatively attributed to the decomposition of the ethylene carbonate electrolyte solvent.

**Figure 3:**
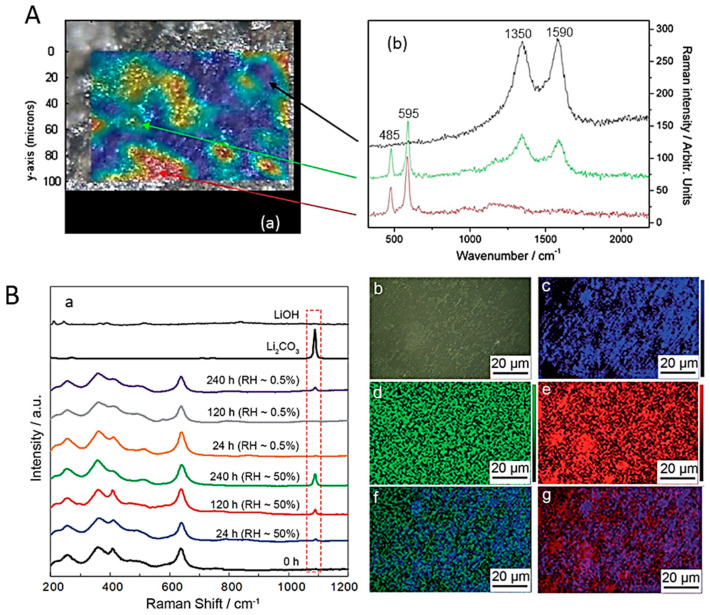
(**A**) Raman map of the LiCoO_2_ cathode composite (85% LiCoO_2_, 10% PVDF, and 5% carbon black) recorded with the laser excitation line at 532 nm. (**a**) Spectral patterns of LiCoO_2_ and carbon are shown in the top-left image and bottom-left image, respectively. (**b**) Raman spectra correspond to the indicated points of the Raman map. Reproduced with permission from [15]. (**B**) Raman analysis of a garnet solid electrolyte Li_7_La_3_Zr_2_O_12_ (LLZO) sample before and after exposure to ambient and dry air (**a**). The dotted line highlights the growth of the Li_2_CO_3_ layer on LLZO as a function of exposure time and RH. Topographic analysis of LLZO exposed to air (RH = 50%) for 240 h. (**b**) Optical image of LLZO, and Raman mapping of LLZO (**c**), LiOH (**d**), Li_2_CO_3_ (**e**), the overlay of LLZO (blue) and LiOH (green) (**f**), and the overlay of LLZO (blue) and Li_2_CO_3_ (red) (**g**) to show the distribution of different phases on the surface. Raman spectroscopy was performed using a 532 nm laser with 100 mW power. Reproduced with permission from [25]. Copyright 2017 Royal Society of Chemistry.

Raman images directly reveal the occurrence of particle size-dependent non-uniform crystallization, phase impurity, etc. Raman mapping provides a powerful means to quantify the state-of-charge (SOC) distribution within LiMO_2_ electrodes by offering a direct, spatially resolved probe of the M-O bonding environment. Because M-O bonds lie at the core of the electrochemical processes in layered LiMO_2_ oxides and dictate their structural stability, Raman imaging enables real-time monitoring of local redox activity and heterogeneities during cycling [23]. Raman mapping collects a spectrum from each position of the sample in a single file (spectral hypercube). In a typical experiment, a large area of the sample is scanned with a 1 µm step size to collect Raman spectra from each pixel (~1 μm × 1 μm). Recorded signals over the area are then displayed as heat maps, rendering intensities by degrees of color [15,24]. Raman mapping, as a result of a spatially resolved Raman analysis of a LiCoO_2_ cathode composite (85% LiCoO_2_, 10% PVdF, and 5% carbon black) covering an area of 100 mm × 150 mm, is shown in Figure 3A. Each Raman spectrum was analyzed based on the intensities of the *A*_1g_ phonon band of LiCoO_2_ at 595 cm^−1^ and the sum of the D- and G-carbon bands. The integrated intensities are superimposed on the microscopic image of the studied region. The mapping results show the heterogeneity of the chemical composition along the LiCoO_2_ composite site. The distribution of LiCoO_2_ and carbon is complementary; there is no indication of PVdF-related Raman signals [15]. Another topographic analysis is illustrated in Figure 3B. The Raman analysis of a garnet solid electrolyte Li_7_La_3_Zr_2_O_12_ (LLZO) sample before and after exposure to ambient and dry air highlights the growth of a Li_2_CO_3_ layer on LLZO as a function of exposure time and RH [25].

**Figure 4:**
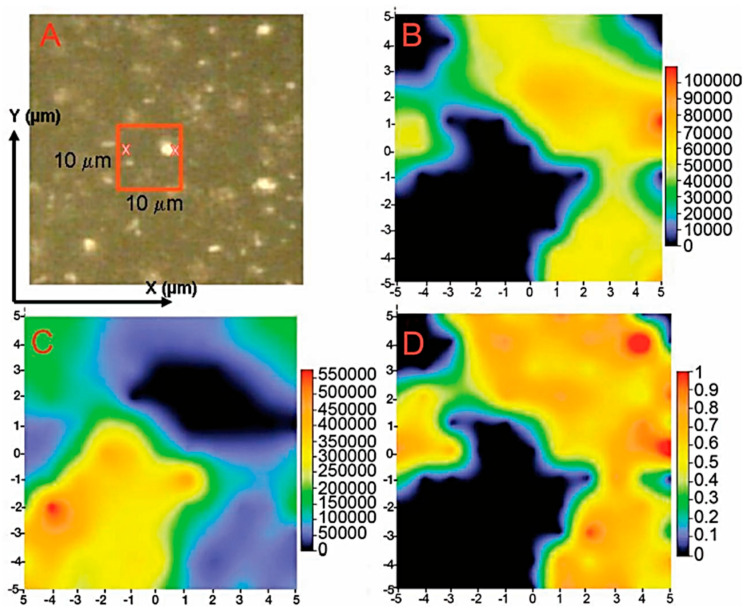
Surface Raman maps of a selected section showing NCA and carbon regions. (**A**) Video image of the studied sample. The red box represents the region selected for Raman mapping. (**B**) Raman map created by plotting the sum of the areas of the NCA Raman bands as a function of electrode position. (**C**) Same as (**B**), but constructed by plotting the sum of the areas of the carbon D1 and G bands as a function of position. (**D**) Raman map of the quantity (*A*_475_/*A*_550_) × (*I*_475_/*I*_550_), which provides a semi-quantitative measure of the NCA particle SOC. Raman experiments were carried out using the 532 nm line laser with a power of ~0.27 mW (~5 kW cm^−2^). The spectrometer was calibrated regularly using the 521 cm^−1^ peak of silicon (111). The spectral resolution was ~1 cm^−1^. Reproduced with permission from [28]. Copyright 2011 Wiley.

The state-of-charge distribution of LiCoO_2_ (LCO) was mapped using a confocal Raman microscope (laser operating at 530.9 nm with a spot of 10 µm^2^ limited to 1–3 mW power) with a resolution of a few micrometers. This allows the intensity, width, and position of the Raman peaks to be determined by constructing the Raman images of the *A*_1g_ mode (595 cm^−1^). The recorded maps contain at least 12 × 12 points, with each point representing a spectrum with a spectral resolution of 10 cm^−1^ [26]. The remarkable potential of Raman imaging and the current-sensing atomic force microscopy (CSAFM) imaging, revealing a significant decrease in the surface electronic conductance of the cathodes in the tested cells, were exploited to understand the local degradation of layered composite cathode materials such as LiNi_0.8_Co_0.15_Al_0.05_O_2_ (NCA) and LiNi_0.33_Mn_0.33_Co_0.33_O_2_ (NMC111) using a 632 nm, 10 mW laser beam [27]. The authors investigated nanoscale changes in surface composition, structure, and state-of-charge (SOC), and also determined the electronic conductivity at the electrode surface. Raman imaging was performed on a 52 × 75 µm^2^ area with a resolution of 0.7. Cathode fluorescence images were recorded in the spectral range 527–691 nm, in response to excitation at 514 nm. Each image is composed of colored pixels, representing the relative intensity of specific Raman peaks and the surface chemical composition, i.e., the presence of active material and carbon additive (acetylene black, graphite, etc.), characterized by the G and D bands of the Raman spectrum. Using micrometer-resolution Raman mapping, Nanda et al. studied the microscopic origin of the SOC related to the local lithium concentration in individual electrode particles and the effective ability of Li^+^ ions to move between redox couples across the geometric boundaries of the cell Li_1−x_(Ni_0.8_Co_0.15_Al_0.05_)O_2_ maintained at different SOCs [28]. Figure 4 shows the results of a mapping experiment performed on a galvanostatically charged NCA electrode at 4.2 V with a 3C charging rate. Fitting of NCA and carbon peaks allowed for the generation of different maps. Figure 4B presents a map of the total area under the NCA spectral bands, and Figure 4C shows a map of the total area under the carbon bands (sum of the areas under the G and D1 bands) as a function of position. A semi-quantitative measurement of the SOC was developed using the product (*A*_475_/*A*_550_) × (*I*_475_/*I*_550_), where *A* and *I* represent the area and intensity (or height) of the peak in the indicated bands, respectively (Figure 4D).

From Raman mapping, kinetics of the crystallization mechanism of materials (i.e., slow crystallization of large particles, but also fast crystallization of small particles) can also be analyzed by fitting with the Kolmogorov–Johnson–Mehl–Avrami (KJMA) relation [29]:

*ϴ* = 1 − exp(−*k*(*t* − *t*_0_)^n^) (16)

where *ϴ* is the crystallinity at each time point (*t*), *k* is the constant depending on nucleation and crystal growth, *t*_0_ is the experimental induction time and n is the nucleation and crystal growth exponent related to the dimensional mechanism. The crystallization rate constant *k*^1/n^ was calculated from the obtained *k* and *n*. For instance, Härtel et al. [30] measured the widths and positions of the internal ν_1_, ν_2_, and ν_3_ bands of SiO_4_ units and the external rotation Raman bands of radiation-damaged zircons between 500 and 1000 °C. The kinetic models, i.e., the KJMA relation and a distributed activation energy model, accurately determine the closure temperature *T*_c_ for damage accumulation for each Raman band.

### 2.5. Resonance Raman Spectroscopy

**Figure 5:**
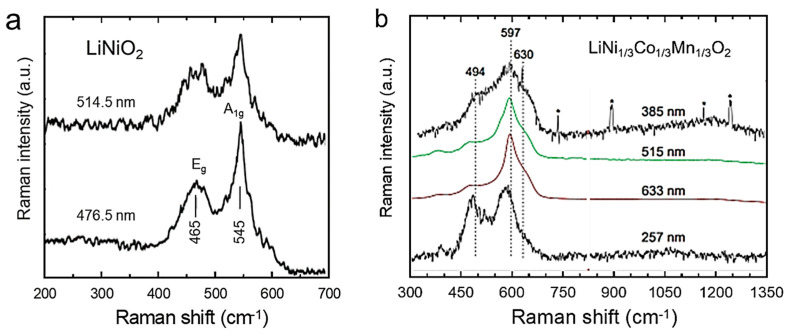
(**a**) Raman spectra of LiNiO_2_ (LNO, synthesized by solid-state reaction) excited at 476.5 and 514.5 nm with power kept below 5 mW. Reproduced from [33]. Copyright 2002 Elsevier. (**b**) Raman spectra of LiNi_1/3_Mn_1/3_Co_1/3_O_2_ (NMC_111_) recorded with UV-visible laser excitations at 257, 385, 515, and 633 nm (low power of 3 mW), spectral resolution of 5 cm^−1^, and wavelength stability of better than 0.5 cm^−1^. Raman peaks were normalized to the *A*_1g_ signal and offset for clarity. Stars (*) indicate parasitic peaks. Reproduced from [39]. Under the terms of the Creative Commons Attribution (CC BY) license.

Resonance Raman spectroscopy (RRS) is an advanced analytical method based on selecting the excitation wavelength to match the energy of an electronic transition. A strong dependence on the laser wavelength allows access to enhanced Raman cross-sections and unique spectral fingerprints of different compounds or components within a material [31,32]. Due to its high sensitivity to phase identification, the resonance Raman effects have been exploited to study the structural complexity of cathode materials for LIBs, such as LiCoO_2_ [15], LiNiO_2_ [33], LiMn_2_O_4_ [34], LiNi_0.8_Co_0.15_Al_0.05_O_2_ [35], LiNi_x_Mn_2−x_O_4_ [36], and LiNi_1/3_Co_1/3_Mn_1/3_O_2_ [37]. RRS measurements can be readily made with a wavelength-tunable excitation laser to meet the resonance conditions for the electronic transitions of the material under study. In practice, however, different lasers with fixed excitation wavelengths are often utilized. Gross and Hess demonstrated the potential of Raman spectroscopy for spatially resolved and in situ analysis of cathode materials. They carried out RRS measurements for the structural characterization of LiCoO_2_-based composite under working conditions of the battery, using three visible excitation wavelengths, i.e., 514.5, 532.0, and 632.8 nm laser line set at 7 mW and at a resolution of 5 cm^−1^ [15]. Julien et al. [33] investigated the electronic structure dependence on the vibrational features of LiNiO_2_, using two different laser line energies (Figure 5a). As expected, Raman peaks appear at the wave number but change in peak intensities when the excitation lines λ = 476.5 and 514.5 nm are used. The increase in the *A*_1g_ mode intensity from *I*(*A*_1g_)/*I*(*E*_g_) = 1.5 to 2.0 with the increase in the laser-line energy provides direct evidence for electronic resonance in LiNiO_2_. This is in agreement with the observation of Aroca et al. [38], who reported an intensity ratio *I*(*A*_1g_)/*I*(*E*_g_) = 0.5 for the excitation at λ = 780 nm.

Heber et al. [39] utilized a UV-beam excitation, which enhanced the fundamental understanding of the NMC111 electrode structure. Initially, laser excitation in the near-UV (at 385 nm) and deep-UV (at 257 nm) regions increased sensitivity to Ni in NMC compared to the visible wavelengths used (at 515 and 633 nm) (Figure 5b). Furthermore, laser excitation at 257 nm allows for the quantification of nickel content in LiNi_x_Co_1−x_O_2_ (and NMC) materials, which is also of great interest for other nickel-containing layered materials.

## 3. Tools for Raman Analysis

### 3.1. Polarization Analysis

**Figure 6:**
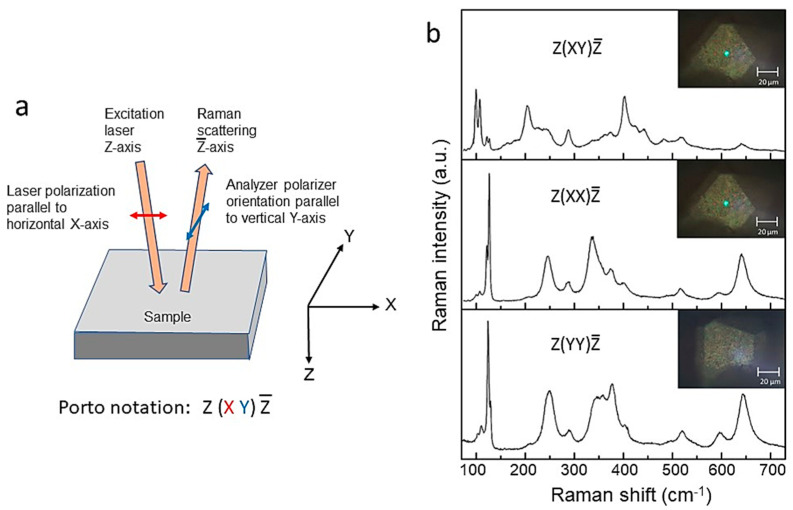
(**a**) Principle of micro-Raman polarization of a crystalline monolith investigated in the backscattering geometry. (**b**) Typical polarized Raman spectra of a tetragonal Li_7_La_3_Zr_2_O_12_ micro-crystal garnet solid electrolyte. Polarization labels refer to the laboratory frame axes *XYZ* (Scott–Porto notation) for both 180° and 90° scattering geometries. The spectra were excited by the 568.2 nm laser line and detected by a CCD (256 × 1024 pixels), cooled at −134 °C by liquid nitrogen. The spectral resolution was about 0.6 cm^−1^/pixel. Reproduced with permission from [41]. Copyright 2013 Elsevier.

In order to derive the symmetry assignment of the Raman-active modes of any material, a careful and detailed polarization analysis of its Raman spectrum must be performed on a single crystal. Porto notation provides a way to record sample orientation with respect to outgoing, post-sample, analyzer orientation, and incident light polarization [40]. The notation includes four terms as follows: The notation includes four terms as *A*(*BC*)*D*, where *A* represents the excitation incident laser axis, *B* is the direction of the excitation polarization, *C* stands for analyzer polarizer orientation, and *D* refers to the Raman scattering axis. The terms beyond the parentheses describe the propagation direction of the outgoing Raman scattering path and the incident laser beam path. The terms within the parentheses define the direction of polarization of the two light paths. For this purpose, the polarized micro-Raman spectra of the crystalline monolith are recorded using two sampling methods: the backscattering geometry and the 90° scattering angle geometry. A micro-manipulator is used to orient the crystal under the objective lens of the microscope and to collect the scattered radiation. The analysis must be performed by recording Raman spectra under three different polarizations. In a typical experiment in 90° scattering geometry, according to Scott and Porto [40], the incident laser beam propagates along one crystallographic axis, the polarization of the incident light coincides with a second axis, and the scattered radiation is collected along the third. Therefore, in the laboratory *XYZ* coordinate system, parallel polarization is denoted *X*(*YY*)*Z*, while cross polarization is denoted *X*(*YX*)*Z*. In the case of backscattering geometry, the four polarization tensors (Scott–Porto notation) investigated by a Raman microscope are *Z*(*XX*)$\bar{Z}$, *Z*(*YY*)$\bar{Z}$, *Z*(*YX*)$\bar{Z}$, and *Z*(*XY*)$\bar{Z}$ (see Figure 6a). For example, the typical micro-Raman spectra recorded under different polarizations from a tetragonal Li_7_La_3_Zr_2_O_12_ micro-crystal garnet in both 180° and 90° scattering geometries are shown in Figure 6b [41]. Polarization labels refer to the laboratory frame axes *XYZ* (Scott–Porto notation).

### 3.2. Curves Fitting

Numerous software packages are dedicated to Raman spectroscopy with key features including baseline subtraction, removal of spectral patterns originating from substrates and solvents, noise reduction, spectral identification, band parameters (position, width, and height), domain size and distribution analysis from Raman images, etc. The curve-fitting relies on the original nonlinear peak-fitting algorithm described by Marquardt and known as the Levenberg–Marquardt method [42]. The fitting of complex band profile curves often appears to be applied in a scientifically inappropriate manner. In particular, the calculation is performed assuming a linear baseline for the spectra and assuming that all Raman lines introduced into the fitting have a mixed Gaussian G(ν)–Lorentzian L(ν) profile (Voigt profile) of the following form:

*S*(ν) = *α G*(ν) + (1 − α)*L*(ν) (17)

where the Gaussian and Lorentzian components are described by the following:

(18) $$L(\nu )=L_{0}\frac{\nu_{L}^{2}}{(\nu -\nu_{0}{)}^{2}+\nu_{L}^{2}}$$

(19) $$G(\nu )=G_{0}\exp\left[-{\left(\frac{\nu -\nu_{0}}{\omega_{G}}\right)}^{2}\right].$$

To understand this G-L line–shape mixing, let us consider one molecule in an excited state. It returns to the ground state within a relaxation called the lifetime *τ_e_* of the excited state. However, in a solid, the molecules are vibrating coherently, but motion and slight differences in vibrational frequencies result in decoherence within a coherence time *τ_c_*. The overall Raman band shape originates from the sum of all the individual vibrations, and the exact vibrational frequency of a particular molecule is controlled by its environment. If τ_c_ << τ_e_, the incoherence sets in rapidly, so dephasing is the dominant energy loss channel. The resulting line shape is Lorentzian (due to the exponential vibrational population relaxation). In the opposite case τ_c_ >> τ_e_, the excited molecule relaxes before incoherence becomes severe. The individual molecules of the solid undergo a statistical distribution of their environments, and the spectral line adopts either a bell curve or a Gaussian profile. Between these limits, interactions prevent extremely rapid movement, but the molecules are not immobilized. Consequently, the two lifetimes can be close, and the spectral line exhibits both Gaussian and Lorentzian characteristics. Equation (17) is an approach to take this mixing into account. The third G-L law gives good results in liquids and gases where the Lorentzian component is dominant. In solids, spectral broadening is commonly described by the convolution of a Gaussian and a Lorentzian function, resulting in the so-called Voigt profile [43]. Unlike the Gaussian–Lorentzian (G-L) mixed profile, the Voigt function allows the Gaussian and Lorentzian linewidths to vary independently, providing a more accurate representation of inhomogeneous and lifetime broadening mechanisms. During curve fitting, the baseline can be modeled as constant, linear, quadratic, or cubic, and is optimized to minimize the residuals while avoiding undesirable artifacts.

**Figure 7:**
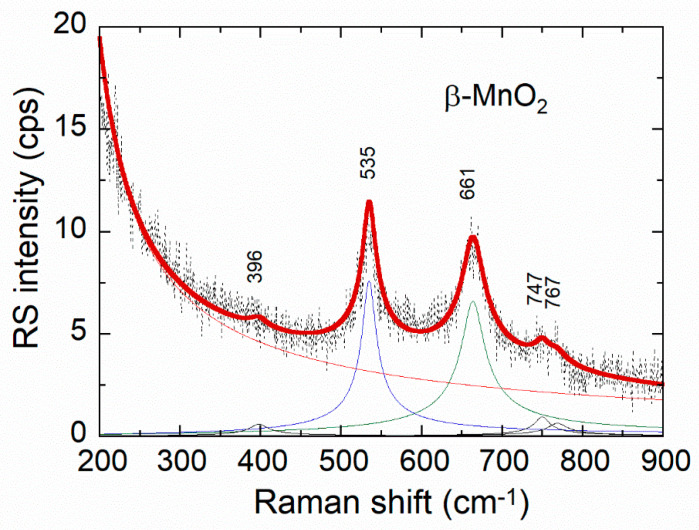
Fitting of the Raman spectrum of β-MnO_2_ using Lorentzian-shaped (LS) bands with χ^2^ = 2.3. The red curve is the reconstructed Raman spectrum, i.e., the sum of LS bands after reducing the noise and subtracting the background with a quadratic baseline.

As an example, we present the curve fitting of Raman bands for the MnO_2_ sample (Figure 7). It is accomplished by the selection of the spectral range 200–900 cm^−1^. After determination of the baseline, the individual Raman band was synthesized using the Lorentzian line-profile, the broadening of which is due to the sample characteristics, i.e., to a disordered structure. Note that the Raman spectrometer is calibrated regularly using the 521 cm^−1^ peak of silicon (111) with a spectral resolution of ~1 cm^−1^.

## 4. Raman and FTIR Analysis of Cathode Materials

In situ and operando Raman and FTIR spectroscopies were widely used to successfully collect data correlated with electrochemical performance results (e.g., capacity retention, Coulombic efficiency) during various steps of battery operation. Raman spectroscopy has proven to be an efficient tool for direct observing electrochemical and structural changes in real time [44]. For example, an operando Raman study combined with electrochemical Butler–Volmer analysis was conducted to investigate the heterogeneous charge-transfer kinetics of the Li-rich γ-Li_1.48_V_2_O_5_ cathode material. Raman spectra were collected at 18 mV intervals, revealing a direct correlation between the shifts in the active vibrational modes *A*_g_, *B*_g_, *A*_u_, and *B*_u_ and the evolution of the Faradaic current at the working electrode. Both the Raman intensity and the Raman shift were subsequently used as proxies for the electrochemical current in a Tafel analysis, enabling the extraction of Butler–Volmer kinetic parameters directly from the spectroscopic data [45].

### 4.1. Layered (Rock-Salt) Structure

**Figure 8:**
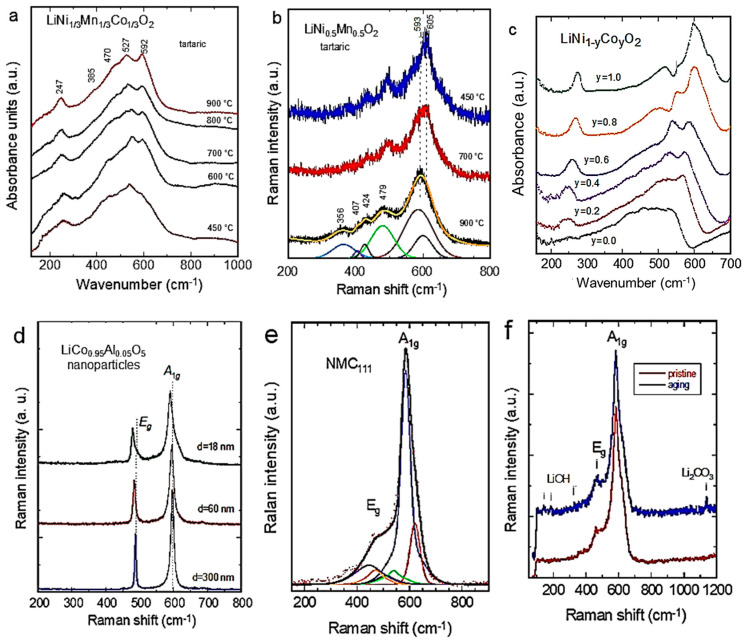
Vibrational features of layered *α*-NaFeO_2_-type cathode materials. (**a**) FTIR spectra of LiNi_1/3_Mn_1/3_Co_1/3_O_2_ vs. sintering temperature. (**b**) Raman spectra of LiNi_0.5_Co_0.5_O_2_ vs. sintering temperature. (**c**) FTIR spectra of LiNi_1−y_Co_y_O_2_ for different amounts of Co dopant (0 ≤ *y* ≤ 1). (**d**) Raman spectra of LiCo_0.95_Al_0.05_O_5_ nanoparticles showing the effect of phonon confinement. (**e**) Raman spectrum of NMC111. The thick line over the raw data is the best fit obtained by decomposition of both the *A*_1g_ and *E*_g_ structures in three Lorentzian bands (dotted and dashed curves) corresponding to the vibrations of the three metal ions with the oxygen. Raman spectra were collected using the 514.5 nm line, low excitation power of 100 W cm^−2^, and resolution of 2 cm^−1^. Reproduced from [49]. Copyright 2010 Elsevier. (**f**) Raman spectra of pristine and aged NMC111 powders after exposure to a humid atmosphere for 24 h. Reproduced from [50]. Copyright 2011 Elsevier.

Layered LiMO_2_ (*M* = Ni, Co, Mn, and mixing of them) oxides, which are lithium intercalation compounds widely used as cathode materials, crystallize with the *α*-NaFeO_2_-type structure (*R*$\bar{3}$*m* space group and spectroscopic $D_{3d}^{5}$ symmetry), so the vibrational modes of *α*-NaFeO_2_ are decomposed as follows:

*Γ* = *A*_1*g*_ + *E*_*g*_ + 2*A*_2*u*_ + 2*E*_*u*_ (20)

where *A*_1*g*_ + *E_g_* are Raman-active modes, which correspond mainly to vibrations of oxygen cages, and 2*A*_2*u*_ + 2*E_u_* are infrared-active modes, which correspond to the stretching and bending modes of metal–oxygen bonds. Figure 8 shows typical Raman and FTIR spectra of several layered materials, i.e., LiNi_1/3_Mn_1/3_Co_1/3_O_2_, LiNi_0.5_Co_0.5_O_2_, LiNi_1−y_Co_y_O_2_ (0 ≤ *y* ≤ 1), and LiCo_0.95_Al_0.05_O_5_. In Figure 8a,c, the FTIR absorption spectra of LiNi_1−y_Co_y_O_2_ powders show the spectral dominance of stretching modes at ν > 500 cm^−1^ and the IR resonant Li-cage mode located between 269 and 234 cm^−1^. The frequency shift in the vibronic modes conforms the Vegard’s law observed by X-ray diffraction [46]. The distribution of the transition-metal ions in the (Ni, Co)O_2_ sheets can also be analyzed from the shape of the FTIR bands, thanks to the predominance of the stretching modes of (Co,Ni)O_6_ octahedra at ca. 500−600 cm^−1^. It should be noted that replacing Ni for Co does not change the space group. This was confirmed by the presence of the two Raman bands (modes *A*_1*g*_ and *E_g_*) of the LiN_1−y_Co_y_O_2_ samples, although the intensities of these peaks are weak, depending on composition and disorder in the cation sublattice.

Let us consider the case of LiN_1−y_Co_y_O_2_ mixed oxides (Figure 8c). Cationic disorder in the (Ni_1−y_Co_y_)O_2_ slabs provokes a frequency shift in the stretching and bending modes, apparently related to cobalt substitution. The frequency shift in the LiO_6_-cage mode has two origins: (i) the slight expansion of the interlayer distance (c_hex_ cell parameter) with increasing temperature, and (ii) the weak mixing of Li-O stretching and O-M-O bending vibrations appearing in the low-wavenumber region. The frequencies of the IR allowed modes at high wavenumbers to vary with the Co content in the LiNi_1−y_Co_y_O_2_ oxides. We observe a quasi-linear variation with *y*, resulting from the one-mode behavior of the LiNi_1−y_Co_y_O_2_ solid solution system. Furthermore, the evolution of the ν_7_-IR band due to the Li-O stretching mode exhibits the characteristic one-mode behavior, with a slight shift towards low wavenumbers for Ni-rich samples. This effect is assigned to the cationic mixing that appears at a low Ni concentration (*y* > 0.3).

The mixed transition-metal LiNi_1/3_Mn_1/3_Co_1/3_O_2_ (named NMC111) is considered as an alternative 4-volt cathode to replace LiCoO_2_ for use in the next generation of LIBs [47,48]. Zhang et al. investigated a series of NMC powders prepared via a wet-chemical route assisted by tartaric acid as a chelating agent. Various acid/metal ion (*R*) ratios have been used to study the influence of this parameter on physical and electrochemical properties of NMCs [49]. It was found that LiNi_1/3_Mn_1/3_Co_1/3_O_2_ sintered at 900 °C for 15 h with *R* = 2 constituted the optimum condition for this synthesis. In this optimized sample, only 1.3% of the nickel ions occupied the Wyckoff 3*b* site of the lithium-ion sublattice. The electrochemical cell provided an initial discharge capacity of 172 mAh g^−1^ in a cut-off potential of 2.8–4.4 V, with a Coulombic efficiency of 93.4%. As the NMC network has three different transition-metal cations, one expects 3*A*_1g_ and 3*E*_g_ Raman-active modes that overlap to give rise to the two broad *A*_1g_ and *E*_g_ structures. The optimal fit to the Raman spectra was then obtained from a prescribed set of three individual Lorentzian-shaped bands for the *A*_1g_ band profile and similarly for the *E*_g_ band profile. Let us take the sample (*R* = 2) as an example (Figure 8e): the *E*_g_ bands are centered at 467, 483, and 510 cm^−1^, and the *A*_1g_ bands are located at 547, 591, and 625 cm^−1^, for *M* = Ni, Co, and Mn, respectively. These positions correspond well to the bands of LiNiO_2_, LiCoO_2_, and *λ*-LiMn_2_O_4_, respectively [1]. Furthermore, after normalization, the band strength (integral of the individual Lorentzian band) of the Ni-O and O-Ni-O vibrations (ν_1_ and ν_4_, respectively) is lower in the *R* = 4 sample than in the *R* = 2 sample. This characteristic is due to the fact that Ni(3*b*) cannot participate in the *A*_1g_ and *E*_g_ modes, and it indicates greater cation mixing between Li^+^ and Ni^2+^ in the *R* = 4 case. It should also be noted that the widths of these modes ν_1_ and ν_4_ are larger in the *R* = 4 case, meaning that the lifetime of these phonons is shorter. This provides further evidence of the greater cationic disorder in this sample, consistent with the structural analysis [49].

The reactivity of a lamellar oxide with water has been tested on NMC111 powders. Figure 8f displays the vibrational patterns of NMC111 powders exposed to ambient atmosphere over 24 h [50]. The Raman spectrum of aged NMC111 shows three additional bands at 144, 189, and 324 cm^−1^, which are attributed to the vibrational modes of LiOH. The high-wavenumber band at 1137 cm^−1^ is assigned to the CO_3_ molecular unit, indicating the presence of Li_2_CO_3_ [51]. This reflects the general trend observed for intercalation compounds, whether lamellar or not: the reaction of lithium with H_2_O at the surface leads to the delithiation of the surface layer, with the lithium involved forming LiOH and Li_2_CO_3_. In their research on LiVO_2_, Manthiram and Goodenough [52] were the first to demonstrate the lithium-ion migration to the sample surface when the particles are exposed to moisture. The subsequent formation of the Li_2_CO_3_ layer has also been observed in other electrodes such as LiNiO_2_ and its analogs LiNi_1−x−y_Co_x_Al_y_O_2_ and in LiFePO_4_. However, the difference between olivine compounds and lamellar compounds lies solely in the thickness of the delithiated layer. Quantitatively measuring this parameter by Raman spectroscopy is difficult. However, this can be performed by other means, notably by analyzing magnetic measurements. The delithiated layer has a thickness of approximately 5 nm in the case of LiFePO_4_ [53], but 10 nm in the case of LiNi_1/3_Mn_1/3_Co_1/3_O_2_, and other layered compounds, such as the oxide LiNi_0.8_Co_0.15_Al_0.05_O_2_ [54]. Consequently, lamellar compounds are more sensitive to moisture than olivine materials. This is due to the degradation of the electrochemical performances induced by these areal chemical reactions with water, and it is mandatory to store the powders in a dry chamber. 

To investigate structural and chemical changes during electrochemical cycling, manganese-based cathode materials have been extensively examined [55]. An O2-type Li_x_[Li_0.2_Mn_0.8_]O_2_ cathode featuring a ribbon-type superlattice was prepared via electrochemical ion exchange, enabling highly reversible cycling performance, as confirmed by Raman spectroscopy, XPS, and impedance measurements. Raman spectra were recorded at various states of charge (SOCs) using a 532 nm excitation line. Upon charging to 4.5 V, the band at 653 cm^−1^, assigned to the *A*_1g_ vibrational mode, undergoes a slight blue shift, reflecting changes in MnO_6_ octahedra associated with anion redox. During discharge to 2 V, the *A*_1g_ band shifts markedly to 609 cm^−1^, indicating a transition from the orthorhombic to the monoclinic phase and a severe distortion of MnO_6_ units during alkali-ion intercalation at low voltage. XPS spectra reveal that the Mn 2p_1/2_ and 2p_3/2_ core-level positions remain essentially unchanged at full charge but shift to lower binding energies upon discharge to 2 V. This result demonstrates that Mn ions contribute to charge compensation primarily during the low-voltage region of the initial discharge process.

### 4.2. Spinel (Cubic) Structure

**Figure 9:**
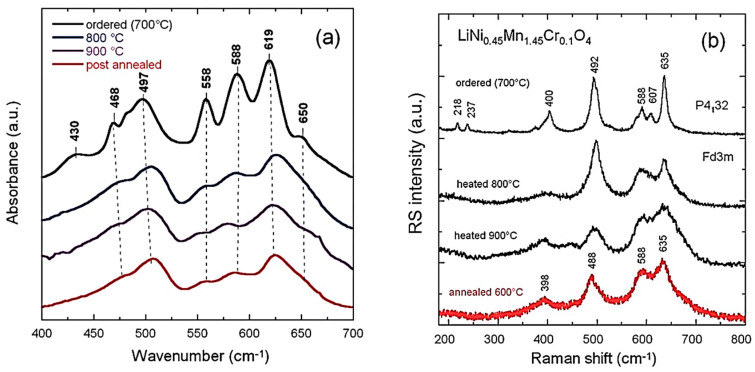
FTIR (**a**) and Raman scattering (**b**) spectra of the LiNi_0.45_Mn_1.45_Cr_0.1_O_4_ samples recorded at different stages of the synthesis: at temperatures in the range 700–900 °C before calcination, and for the final sample with annealing at 600 °C for 48 h. Raman spectroscopy was performed using a 532 nm laser with 10 mW of power. Reproduced from [57]. Copyright 2014 Royal Society of Chemistry.

LiNi_x_Mn_2−x_O_4_ (LNM) spinel oxides, which belong to the 5 V high-voltage class of cathodes, are known to be promising positive electrodes for Li-ion batteries [56]. The LNM structure (*Fd*$\bar{3}$*M* S.G.) consists of Li and Mn cations occupying tetrahedral (8*a*) and octahedral (16*d*) sites, respectively, within a close-packed cubic lattice of oxygen atoms (32*e* sites). LNM crystallizes in two crystallographic forms: the face-centered spinel (*Fd*$\bar{3}$*M* S.G.), noted “disordered spinel”, and the simple cubic phase (*P*4_3_32 S.G.), noted “ordered spinel”. Numerous studies have shown that the growth of the ordered/disordered phase of LNM is dependent on the synthesis process and the synthesis conditions: nature of the precursor, sintering temperature, and rich-O_2_ atmosphere; acute control of the cooling rate immediately after calcination is also effective. The vibrational spectroscopy proved very useful to evaluate the degree of cation disorder. Figure 9 presents the FTIR and Raman spectra of the LiNi_0.45_Mn_1.45_Cr_0.1_O_4_ electrode materials prepared at different stages of the synthesis, i.e., for temperatures up to 900 °C before calcination, and for the final sample, after annealing at 600 °C for 48 h in air [57]. For these samples, the Ni–O stretching vibronic mode at 588 cm^−1^ and Mn-O at 619 cm^−1^ dominate the spectra. The characteristic bands (around 430, 468, 558, and 650 cm^−1^) corresponding to the ordered structure of the cations show a high intensity only in the spectrum of the sample annealed at 700 °C, and they are almost absent in the spectra of the other specimens. This result confirms that the order/disorder transition occurs only at 700 °C [58]. A semi-quantitative evaluation of the degree of order of the cations in the spinel phase can further be characterized by the ratio of the intensity of the main band at 619 cm^−1^ to that of the main additional band present only in the ordered phase (*P*4_3_32 S.G.) at 550 cm^−1^: the larger this ratio, the larger the cation disorder in the molecular range probed by the FTIR spectroscopy. Comparison between the FTIR spectrum of the Cr-doped LNM specimen and that of the initial sample annealed at 600 °C (Figure 9a) shows that the initial material exhibits a more ordered phase than the sample doped with chromium. Despite the disorder of the cations in the sample with the *O*_h_^7^ spectroscopic symmetry, the FTIR spectrum shows well-resolved stretching modes, unlike a typical LiMn_2_O_4_ spinel, which exhibits broad and overlapping bands. These results can be understood in terms of short-range Ni^2+^ and Mn^4+^ cation ordering on the octahedral sites. The degree of Ni/Mn order on the surface layers of the three samples was also investigated with Raman scattering spectroscopy. Figure 9b shows the typical Raman spectra of crystallized Li[Ni_0.5_Mn_1.5_]O_4_ samples [57].

The octahedral structural units Mn(Ni)O_6_ exhibit *O*_h_ symmetry. The peak at 625 cm^−1^, attributed to the symmetric Mn-O stretching mode of MnO_6_ octahedra in Li[Mn_2_]O_4_, is shifted towards higher wavenumbers (at 635 cm^−1^). The new bands at 399 and 490 cm^−1^ are intense and can, therefore, be unambiguously attributed to the Ni-O stretching mode. Two factors contribute to the frequency shift in this mode: the increase in the average valence state of the Mn ions and the decrease in the unit-cell volume. None of the characteristic peaks of the ordering of Ni(II) and Mn(IV) in space group *P*4_3_32, namely at 218, 237, and 607 cm^−1^, were detected, which confirms the disorder of Ni(II) and Mn(IV) on the 16*d* sites of spinel in the LNM samples [59].

### 4.3. Phosphate (Olivine) Structure

Three-dimensional (3D) materials containing (PO_4_)^3−^ anions in their structure have attracted particular attention due to the high energy density, cost-effectiveness, and good environmental compatibility of their basic constituents. Examples include LiFePO_4_, LiFeP_2_O_7_, Li_3_Fe_2_(PO_4_)_3_, and Li_3_V_2_(PO_4_)_3_ crystallizing with an olivine-like or Nasicon-like lattice [60,61]. These compounds have several advantages compared to that of conventional lithium metal oxides, which are as follows: high redox potentials, rapid Li^+^ ion transport, and enhanced thermal stability. These lattices, containing an interconnected interstitial space, are potentially fast ionic conductors, especially during substitution of the bulkiest polyanion. They help to stabilize the structure and enlarge the 3D tunnel network to allow for fast ion migration.

The olivine-like structure of LiFePO_4_ (LFP) consists of a hexagonal close-packed (*hcp*) lattice of oxygen atoms. Li^+^ and *M*^2+^ cations occupy half of the octahedral sites, while P^5+^ cations occupy one-eighth of the tetrahedral sites [62,63]. This structure can be described as a series of chains (oriented along the *c*-axis) of edge-sharing FeO_6_ octahedra, cross-linked by the PO_4_ groups, thus forming a 3D lattice. Tunnels perpendicular to the [010] and [001] directions contain octahedrally coordinated Li^+^ cations (oriented along the *b*-axis). Li^+^ ions are mobile in these cavities. These compounds generally crystallize in the orthorhombic (*PnMa* S.G.) system.

**Figure 10:**
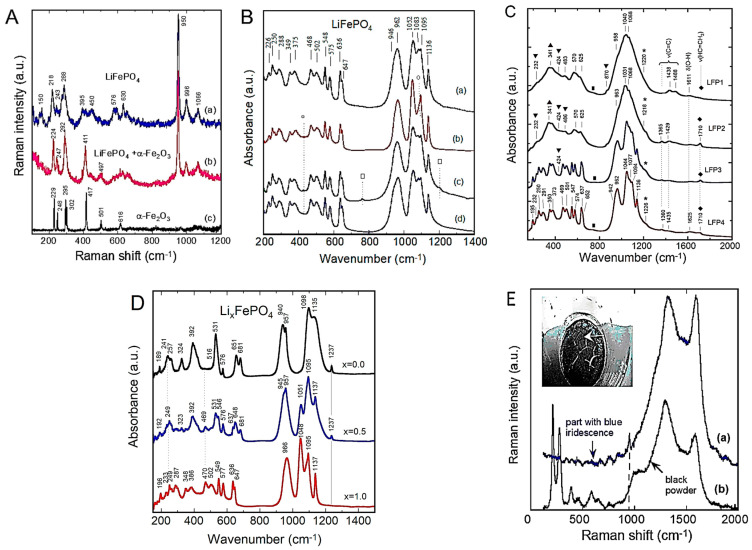
Vibrational features of LiFePO_4_ (LFP) olivine cathode material. (**A**) Raman spectra of (a) pure LFP, (b) α-Fe_2_O_3_-containing LFP, and (c) pure α-Fe_2_O_3_. The spectrum of pure α-Fe_2_O_3_ is shown for comparison. (**B**) FTIR of LFP samples synthesized using different wet-chemistry methods: (a) acetate-based sol–gel method, (b) multistep grinding process, (c) gelation of iron nitrate, and (d) solid-state reaction. Impurities such as magnetite and pyrophosphate are detected. Extrinsic vibrations are marked by different symbols: ☐ (P_2_O_7_)^4−^ and ◯ Li_3_PO_4_. (**C**) FTIR absorption spectra of lithium iron phosphate materials synthesized through polymeric precursor. LFP powders were dispersed in anhydrous CsI in a 300:1 ratio for easy measurements in the far-infrared region. Spectra of LFP3 (*T*_a_ = 300 °C, 4 h) and LFP4 (*T*_a_ = 400 °C, 24 h) powders display IR bands characteristic of the LiFePO_4_ olivine lattice. Extrinsic vibrations are marked by different symbols: ■, LiFeP_2_O_7_; *, Li_3_Fe_2_(PO_4_)_3_; ▼, Li_3_PO_4_; ▲, FePO_4_; ◆, (–HC=CH_2_) vinyl group of the polymer; and ●, δ(O-H) bending mode coming from the FePO_4_∙2H_2_O precursor, while other bands are associated with ν(C-C) vibrations. FTIR spectra were recorded at a spectral resolution of 2 cm^−1^. Reproduced from [64]. Copyright 2006 Elsevier. (**D**) FTIR spectra of LFP electrode at different Li contents (*x* = 1.0, 0.5, and 0.0). The continuous vertical lines point out the position of the band, which only exists in FePO_4_. The broken vertical lines point out the bands, which only exist in LiFePO_4_. Reproduced from [64]. Copyright 2007 American Chemical Society. (**E**) Raman spectrum of the deposit formed after LFP powders were aged in water. (**a**) and (**b**) spectra refer to the black powder and the blue iridescence formed upon aging. The vertical broken line points to the position of the stretching mode of PO_4_ units. Reproduced from [53]. Copyright 2008 Elsevier.

Figure 10A shows the Raman spectra of a pure LFP sample synthesized by solid-state reaction (a) and a sample containing α-Fe_2_O_3_ impurity. Since the structure of phospho-olivine consists of octahedral LiO_6_ and FeO_6_ units bonded to (PO_4_)^3−^ polyanions, the local cationic arrangement can be studied using factor group analysis and a molecular vibration model. The internal vibrations of phospho-olivine materials can be deduced from the fundamental (PO_4_)^3−^ (*T_d_* symmetry) ν_1_–ν_4_ modes. Furthermore, the stretching and bending mode regions are very distinct. As expected, the fundamental vibrations of the PO_4_^3−^ polyanions dominate the vibronic spectra, which are decomposed into numerous components due to the correlation effect induced by coupling with the Fe-O units present in the structure. In the region of the internal modes of the phosphate anion (region of high-wavenumber), the symmetric stretching mode at ν_1_ = 950 cm^−1^, the doublet ν_2_ = 410–470 cm^−1^, and the triplets ν_3_ and ν_4_ in the regions 1005–1085 cm^−1^ and 580–640 cm^−1^, are identified. Remarkably, the Raman spectrum shows that the intensity of the symmetric bending mode ν_2_ is lower than that of the antisymmetric bending mode ν_4_, which is unusual.

Figure 10B displays the FTIR spectra of four LFP samples synthesized by various wet-chemical methods: (a) acetate-based sol–gel method; (b) multistep grinding process; (c) gelation of iron nitrate; and (d) solid-state reaction. The FTIR spectrum of sample (a) involves only the band characteristics of LiFePO_4_ with the strong stretching modes (ν_1_ and ν_3_) corresponding to the symmetric and antisymmetric modes of the P-O bonds in the range of 945–1140 cm^−1^. Other FTIR spectra display extra bands due to defects and impurities such as Fe_2_P, α-Fe_2_O_3_, and inclusion of the FePO_4_ phase. The vanishing of the 292 cm^−1^ band attributed to the Li-O vibration is the fingerprint of such a phase inclusion. The structure at the molecular size scale of LFP materials synthesized through FePO_4_(H_2_O)_2_ + Li_2_CO_3_ mixed with polymeric carbon additive (i.e., polyethylene-block-poly(ethylene glycol) 50% ethylene oxide) has been investigated by FTIR absorption experiments (Figure 10C) [63,64,65]. LFP1 product synthesized with carbon black heated at 400 °C for 4 h exhibits an amorphous structure. In the highest-resolution FTIR spectrum of the LFP4 sample (polymeric additive; heated at 400 °C for 24 h), the vibrations of the PO_4_^3−^ units are broken down into numerous components due to coupling with Fe-O units. This richer spectrum indicates the good crystallization of this sample. Indeed, the frequencies of the internal modes in the LFP4 sample spectrum are consistent with those of LiFePO_4_ olivine.

FTIR spectra of LFP samples with three different Li contents (*x* = 0.0, 0.5, and 1.0) are shown in Figure 10C. The FTIR spectrum of a delithiated LFP sample has also been reported by Burba and Frech [66] and by Lucas et al. [67]. Furthermore, the notation used in this previous work is Li*_x_*FePO_4_ to denote partially delithiated samples, but it should be read as *x*LiFePO_4_-(1−*x*)FePO_4_ as in the present work. Since samples are bi-phasic, as is the case for the sample *x* = 0.5 specimen. Juxtaposing the spectra facilitates vibration assignment. During the delithiation process, the main characteristic is that the peak frequencies exhibit only a small shift (of a few cm^−1^) due to the modification of the lattice parameters, and also, below 600 cm^−1^, there is a change in the valence state of iron. This is the case for the external modes, or lattice vibrations occurring below 400 cm^−1^. By attempting to follow the evolution of the frequencies of these modes, a correspondence of the lines (in cm^−1^) can be established from *x* = 1 to *x* = 0: 386→392, 348→324, 287→257, 249→241, and 196→189. This confirms that these modes are mainly translational and vibrational movements of oxo-anions (PO_4_)^3−^ and translational movements of Fe^2−^ ions. However, an additional spectral line is observed at 233 cm^−1^ only in samples with *x* = 0, and it is associated with the vibration of the lithium-ion, i.e., the vibration of Li in its octahedral cage. To identify this mode, we note that the ^6^Li-^7^Li isotopic substitution in lithium-containing transition-metal dioxides has shown that the far-infrared peak between 200 and 300 cm^−1^ is the typical asymmetric stretching mode of LiO_6_ [5]. Particularly, in similar *O_h_* configurations, this mode was observed at 260 cm^−1^ in LiCoO_2_ and at 240 cm^−1^ in LiNiO_2_ [44]. The line at 233 cm^−1^ in the LFP sample with *x* = 0, shifted by only a few cm^−1^ compared to LiNiO_2_, corresponds to the same Li-cage mode of the lithium ions, namely, the translational vibration of lithium ions within the cage formed by the six nearest oxygen atoms. Above 400 cm^−1^, the bands correspond to the external modes associated with the intramolecular vibrations of the PO_4_ and FeO_6_ units. In the spectral region of FeO_6_ vibrations, two modes at 636 and 647 cm^−1^ (for *x* = 1) are displaced to 651 and 681 cm^−1^ (for *x* = 0) after lithium extraction. In the intermediate spectral region 400−600 cm^−1^, the bands at 577, 549, and 502 cm^−1^ (for *x* = 1) can be considered shifted to 576, 531, and 516 cm^−1^, corresponding to the (ν_2_–ν_4_) bending modes of the (PO_4_)^3−^ oxo-anions. Furthermore, an additional band appears at 470 cm^−1^ in the spectrum of the *x* = 0 samples, which is also assigned to the (ν_2_–ν_4_) mode related to the motion of lithium ions. Conversely, an additional band is clearly visible at 1237 cm^−1^ in the FePO_4_ spectrum, which is absent in LiFePO_4_. Note that, in prior work, this additional pattern was also observed in the FTIR spectrum of FePO_4_ [68]. Attempting to identify this mode with phosphate ion complexes, it is worth noting that complexes such as (P_2_O_7_)^4−^ and (P_3_O_10_)^5−^ are absent from LFP material. (P_2_O_7_)^4−^ and (P_3_O_10_)^5−^ ions would generate vibrational modes in the 700–900 cm^−1^ region (spectral gap), where no pattern is detected. On the other hand, it is assumed that this mode is linked to a vibrational mode of PO_3_. Indeed, the lantern units present in the Nasicon phase exhibit infrared bands in the 1150−1250 cm^−1^ region, assigned to the stretching modes of the terminal PO_3_ units, and characteristic of the FTIR spectrum of Li_3_Fe_2_(PO_4_)_3_ [69].

The effect of exposing carbon-coated LiFePO_4_ particles (synthesized via solid-state reaction (SSR)) to water was investigated. Upon immersion of the particles in water, the bath contained a floating portion and a sinking portion. The floating portion was removed before evaporation of the solution, so that the deposit after evaporation consisted of the settled sample and the portion dissolved in water. This deposit was analyzed by Raman spectroscopy (Figure 10E) [66]. Its color is almost black with blue highlights in places. The black color originates from carbon suspended in the water. The presence of these carbon particles suspended in the solution was evidenced by the turbidity of the colored solution in which the SSR sample was immersed. During drying, this suspended carbon settled to the bottom of the container, forming a black crust that is responsible for the black color of the deposit. Blue iridescence is also observed on the surface of iron after phosphatizing, an industrial process used to passivate the surface of iron compounds with a thin layer of Fe(H_2_PO_4_)_2_. In this case, it is attributed to the diffraction of light on the ultra-thin layer. The same phenomenon is likely responsible for this iridescence observed here. Indeed, Fe(H_2_PO_4_)_2_ is soluble in water. This analysis is supported by Raman spectroscopy, a remarkable tool for detecting the presence of carbon, thanks to the two characteristic Raman bands located between 1200 and 1700 cm^−1^. The Raman spectra of the black/blue deposits have been recorded using a He-Ne laser as the excitation source (λ = 632.8 nm). While the intrinsic spectrum of LiFePO_4_ is dominated by the peak at 960 cm^−1^ associated with the stretching mode of the PO_4_ group, the Raman spectrum of the portion exhibiting blue iridescence is dominated by the two characteristic carbon bands. The pattern centered at 960 cm^−1^, however, is clearly visible, indicating that this part of the material also contains phosphate. The Raman spectrum of the black portion, lacking blue iridescence, again exhibits the dominant bands characteristic of carbon. Conversely, the absence of structure at 960 cm^−1^ confirms that the blue iridescence is linked to the presence of phosphate and a phosphatation effect. Furthermore, three additional structures are visible at lower frequencies (398, 263, and 219 cm^−1^) that are characteristic of lithium hydroxide monohydrate LiOH·H_2_O [70]. A broad band is observed around 1070 cm^−1^, which is also present in the region with blue iridescence. This broad band was also detected in the Raman spectrum of molten LiOH and is attributed to the vibration of the CO_3_ molecular unit, thus confirming the presence of Li_2_CO_3_ in addition to the lithium hydroxide.

## 5. Raman and FTIR Analysis of Anode Materials

### 5.1. Nano-Carbon Anode

Raman spectroscopy is a sensitive method to identify different forms of carbonaceous materials [71,72] and for characterizing the structural perfection and organization of carbon, graphite, and carbon fibers [73,74,75,76,77,78]. As a non-destructive technique, it is efficient for the monitoring of the stress and strain in graphite and carbon fibers, as well as in carbon-containing SiC fibers produced by polymer precursors [79], together with the investigation of the Li intercalation process in carbonaceous anodes [80]. The characteristics of the Raman spectrum, i.e., position, width, and intensity of both carbon bands, are sensitive to the structural disorder in the carbon structure. Any difference in the Raman band shape of both carbon bands (named D and G in the range 1300−1600 cm^−1^) reflects changes in the carbon microstructure [81]. Therefore, it is important to understand the curve-fitting procedures used for the comparison of band positions and bandwidths. Since the Raman bands of carbon from the coating are overlapping and relatively broad, determining their position and width can be influenced by the curve-fitting procedure employed, particularly the number of bands selected and the function used to fit them. Due to the asymmetric nature of the G band, which exhibits tails towards lower wavenumbers, it is often necessary to choose additional bands to optimize the full-spectrum fit.

**Figure 11:**
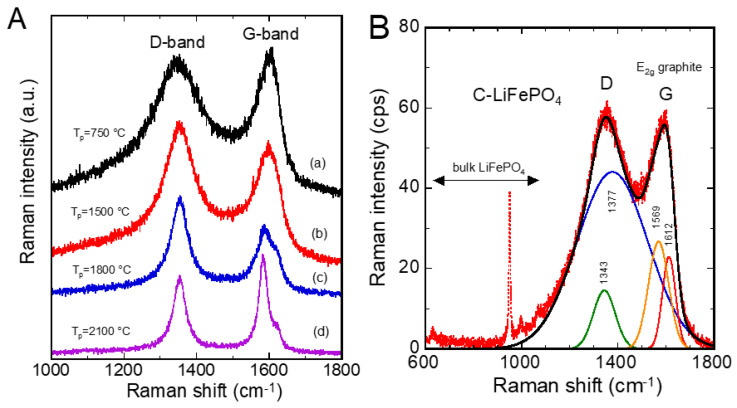
(**A**) Raman spectra showing the shape evolution of the D and G bands of carbon synthesized at various pyrolysis temperatures, *T_p_*. (a) 750 °C, (b) 1500 °C, (c) 1800 °C, and (d) 2100 °C. (**B**) Raman spectrum recorded using the 514.5 nm laser line of a carbon-coated LiFePO_4_ sample showing the symmetric stretching mode of (PO_4_)^3−^ anions at 940 cm^−1^. Reproduced from [81]. Copyright 2006 AIP.

Figure 11A presents the Raman spectra of carbon samples prepared at various pyrolysis temperatures, *T_p_*, showing the evolution of the shape of the D and G bands. The intensity ratio, *I*_D_/*I*_G_, of the bands for the carbon fired at *T_p_* = 750 °C indicates that particles of this carbon have an average size of approximately 10 nm. This is consistent with micro-structural analyses performed by TEM [82]. Figure 11B shows the Raman spectrum of the carbon-coated LiFePO_4_ sample in the range 600–1800 cm^−1^, recorded with the laser line at 514.5 nm. The symmetric stretching mode of (PO_4_)^3−^ anions is pointed at 940 cm^−1^. The carbon spectrum was fitted in various ways: a Lorentzian fit for the D band and two Gaussian fits for the asymmetrical G band at ~1570 and 1610 cm^−1^ [81]. Note that Robertson et al. [78] tested a Lorentzian fit for the D band and an asymmetrical Breit–Wigner–Fano (BWF) fit for the G band. The BWF profile is defined as follows:

(21) $$I\left(\omega \right)=I_{o}\frac{{\left[1+(\omega -\omega_{BWF})/q\Gamma \right]}^{2}}{1+{\left[\left(\omega -\omega_{BWF}\right)/q\Gamma \right]}^{2}}$$

where 1/*q* represents a parameter measuring the interaction between the phonon and the continuum of electronic states in a metal (plasmons being the electronic interactions in the coupling), and *Γ* represents the damping factor. The BWF profile is, therefore, justified only in the case of highly conductive carbon, for example, graphite [80], but not in the case of carbon coated on the particle surface of electrodes. Such a carbonaceous material is poorly crystallized or amorphous coke, a less conductive variety of carbon than graphite [77]. In the fitting presented in Figure 11B, the D and G bands were selected separately using a Lorentzian function on a linear baseline, in accordance with previous studies [73]. It has been demonstrated that the D band increases with disorder or decreases with crystal size, while the *I_D_*/*I_G_* ratio is inversely proportional to the average crystallite size (*L_α_*) for disordered graphite sizing in the range 2–300 nm [20]. The *I_D_*/*I_G_* ratio is expressed by the following equation:

(22) $$\frac{I_{D}}{I_{G}}=\frac{C\left(\lambda \right)}{L_{\alpha }}$$

where *C*(*λ*) is a function of the excitation radiation wavelength. It is equal to 4.4 nm for incident laser wavelengths of 488 and 514 nm [20], and 5.8 nm for an incident laser of 633 nm [82].

**Figure 12:**
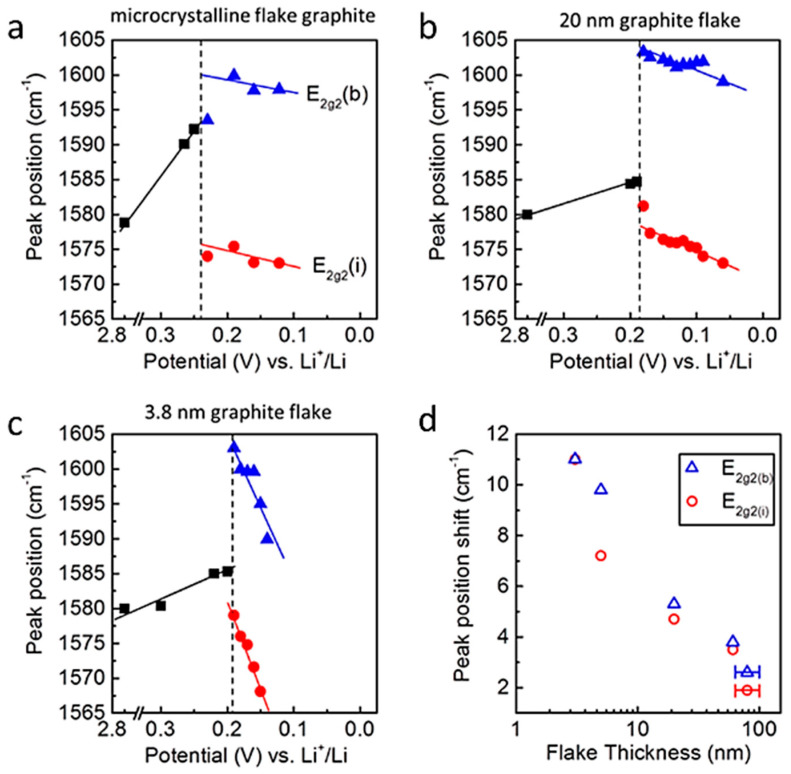
(**a**–**c**) Peak position of the G band during intercalation as a function of the cell potential for microcrystalline flake graphite. (**a**), 20 nm graphite flakes (**b**), and 3.8 nm graphite flakes (**c**). The dashed line indicates the splitting occurrence. A clear split of the G band to E_2g2_(i) (blue symbols) and E_2g2_(b) (red symbols) modes at around 0.22 V was preceded by an upshift in the G band frequency (black symbols). (**d**) Comparison of the Raman peak shift in the split G band for graphite flakes with different thicknesses. Reproduced from [83]. Copyright 2016 under a Creative Commons Attribution (CC-BY) license.

In situ Raman spectroelectrochemical studies of Li intercalation into graphite flakes with different thicknesses ranging from 1.7 nm (3 graphene layers) to 61 nm (ca. 178 layers) were reported by Zou et al. [83]. The effect of intercalation was observed under potential control via in situ optical microscopy and Raman spectroscopy. As the thickness of the graphite sheets decreased, a Raman response indicative of an increase in tensile stress along the graphene sheet was observed in the early stages of intercalation (Figure 12). The progressive shift towards lower wavenumbers of the inner and edge modes of the split G band, i.e., the *E*_2g_^2^(i) and *E*_2g_^2^(b) modes, was due to a weakening of the C-C bond. The Raman spectra were interpreted in terms of intercalation-induced stress, which could accelerate the aging of the carbon and reduce its long-term lifespan.

### 5.2. Nano-Silicon Anode

**Figure 13:**
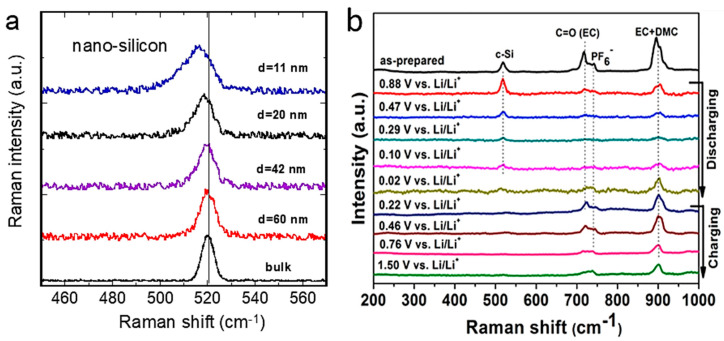
(**a**) Experimental micro-Raman scattering spectra of Si nanoparticles recorded using the laser line *λ*_0_ = 514.5 nm. The evolution of the first-order 520 cm^−1^ Raman peak of the nanoparticle is compared with the spectrum of bulk Si{001}. The vertical line indicates the phonon line of bulk Si. (**b**) In situ Raman spectra acquired from the electrochemically active and inactive points of the Si-based electrode (active mass loading is about 4–5 mg cm^−2^). Dashed vertical lines indicate peaks associated with c-Si, solvents, and PF_6_^−^ anion. Micro-Raman spectra were collected with laser wavelength of 532 nm and 1 μm × 1 μm spatial resolution. Reproduced from [86]. Copyright 2015 Elsevier.

Silicon is a promising material as a negative electrode for LIBs because of its high theoretical energy density of about 4200 mAh g^−1^, ten times greater than that of graphite (372 mAh g^−1^). It has a relatively low operating potential of ~0.5 V vs. Li^+^/Li. However, silicon pulverization due to volume expansion during lithium-ion insertion leads to rapid capacity degradation during charge/discharge cycles. Various nanostructured forms of silicon exhibit promising potential for addressing this issue, including nanocrystals, nanocomposites with carbon or other lithium-inactive phases, nanoporous materials, nanowires, agglomerated Si nanotubes, and thin films. Figure 13a shows the results of Raman microspectroscopy of Si nanoparticles. The shape of the monophononic band 520 cm^−1^ is shown as a function of particle size. As expected, this figure highlights a slight shift towards lower frequencies in the silicon Raman peak at ~520 cm^−1^ and an asymmetric broadening towards lower wavenumbers when the silicon particle size reaches the nanoscale, due to phonon confinement. These complex changes can be explained by the Richter model. The quantum confinement effect of phonons, inducing a relaxation of the Raman selection rule ***q*** = 0, allows the participation of phonons far from the center of the Brillouin-zone, i.e., at point U. It is worth noting that the quantum confinement effect is only effectively observable for structures in which at least one of the three principal dimensions is less than ~20 nm [84,85]. When the Si nanoparticles formed in this study have a larger diameter (*L* ≥ 40 nm), no significant shift towards lower frequencies was observed. It is also clear (Figure 13) that a nanoparticle with an *L* = 42 nm exhibits a very weak phonon confinement signature, i.e., the line shape is almost Lorentzian. Finally, a broadened and slightly asymmetric single-phonon Raman peak is observed at 517 cm^−1^, with a full-width-at-half-maximum (FWHM) of 12 cm^−1^ for nanoparticles with a diameter of 11 nm. Moreover, the first-order Raman intensity is of a similar order of magnitude to that of bulk silicon, as illustrated in Figure 13 [86].

Figure 13b shows in situ Raman spectra acquired from the electrochemically active and inactive points of a crystalline silicon (c-Si) electrode (active mass loading is about 4–5 mg cm^−2^) in an experiment using carbonate electrolyte and LiPF_6_ salt [86]. The vertical dashed lines indicate the position of the Raman band at~520 cm^−1^ characteristic of crystalline silicon (c-Si) and the out-of-plane deformation mode of the O=C ring of ethylene carbonate (EC) at ~714 cm^−1^. The fully symmetrical vibration of the PF_6_^−^ anion in solution was observed at approximately 741 cm^−1^. Significant changes are observed below a potential of 0.88 V during lithiation, indicating the electrochemical activity of these areas: a substantial decrease in signal intensity at 520 cm^−1^ was observed at 0.47 V, and the Raman signal associated with c-Si decreased continuously as the potential decreased from 0.29 to 0.02 V. This effect has been attributed to the progressive formation of amorphous Li_x_Si compounds.

Operando Raman spectroscopy combined with synchrotron XRD was used to study stress evolution in the anodes of a Li-ion battery composed of crystalline silicon nanoparticles [87]. The internal structure of the nanoparticles was evaluated during two discharge/charge cycles by analyzing the intensity and position of diffraction peaks and Raman TO-LO phonons of silicon. Under capacity-limited conditions, the lithiation/delithiation process of silicon induces the formation of particles with a “crystalline core-amorphous shell”, resulting in a progressive decrease in core size, as well as compressive/tensile stress sequences due to the stress applied by the shell. In particular, different sequences are observed during the first and the second cycles, due to different lithiation reactions. Krause and co-workers investigated the first lithiation/delithiation cycle of carbon-coated silicon nanowires using in situ Raman spectroscopy [88]. It was found that, as the lithiation of silicon takes place below 0.2 V, the Si signal drops, due to the formation of a thick solid electrolyte interphase (SEI) layer instead of the reaction of silicon with lithium; a signal at 1859 cm^−1^ appears almost simultaneously with the drop of the Si signal, which is attributed to a component of the SEI.

The electrical conductivity, impedance response, and Raman spectra of amorphous silicon (a-Si) anodes were monitored in situ during lithium insertion and extraction. A pronounced decrease in Raman intensity—followed by its recovery near 0.523 V vs. Li^+^/Li—was observed. This behavior is attributed to changes in the optical skin depth of a-Si associated with the formation and subsequent decomposition of the Li-Si alloy [19].

### 5.3. Titanate-Based Anodes

**Figure 14:**
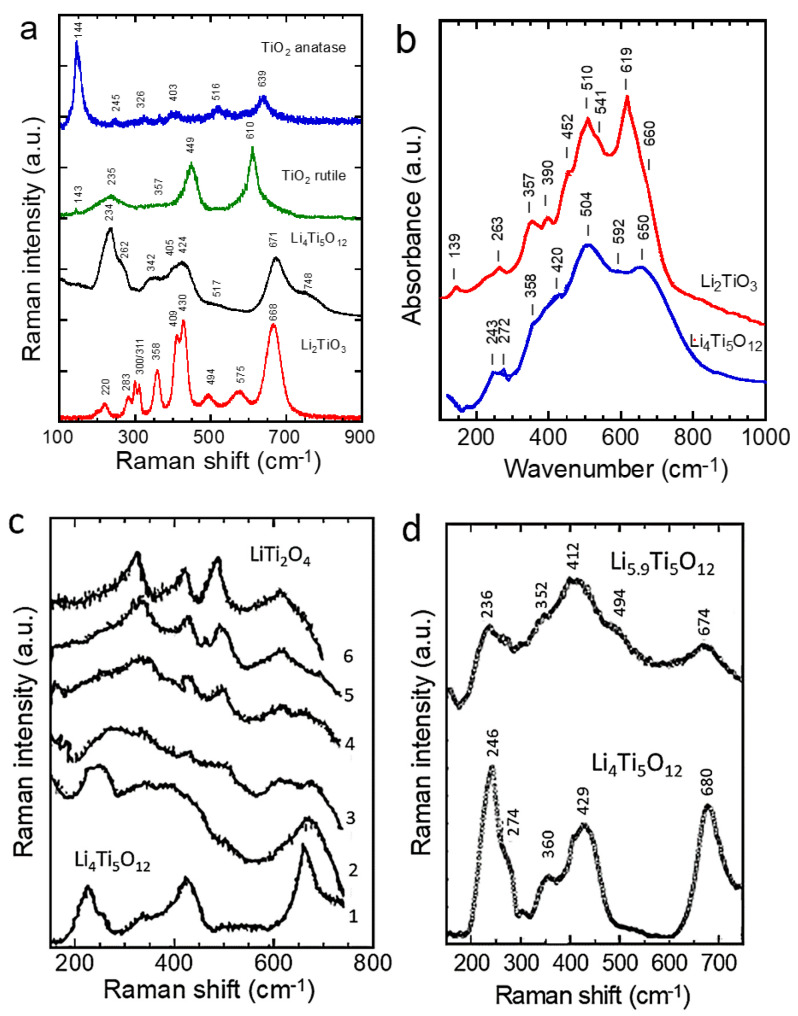
(**a**) Raman spectra of titanates: anatase TiO_2_, rutile TiO_2_, spinel Li_4_Ti_5_O_12_, and monoclinic β-Li_2_TiO_3_. (**b**) FTIR spectra of Li_4_Ti_5_O_12_ and β-Li_2_TiO_3_ recorded in the spectral range 100–1000 cm^−1^ at 2 cm^−1^ spectral resolution. (**c**) Raman spectra of polycrystalline Li_1+x_Ti_2−x_O_4_ powders showing the end members (1) *x* = 1/3 (Li_4/3_Ti_5/3_O_4_) and (6) *x* = 0 (LiTi_2_O_4_). Excitation used the 514.5 nm line radiation. Reprinted with permission from [95]. Copyright 1994 Elsevier. (**d**) Raman spectra of pristine and chemically lithiated Li_4_Ti_5_O_12_. Reproduced with permission from [96]. Copyright 2000 American Chemical Society.

Titanium oxides, including TiO_2_, LiTi_2_O_4_, Li_4_Ti_5_O_12_, and Li_2_TiO_3_, are attractive materials due to their Ti^4+^/Ti^3+^ redox couple working approximatively 1.5 V vs. Li^+^/Li [89,90]. Figure 14a–d presents the Raman and FTIR patterns of these Ti-based compounds [91,92,93,94,95,96]. TiO_2_ crystallizes mainly in two polymorphs: the tetragonal anatase TiO_2_ (*I*41/*amd* S.G.) and rutile TiO_2_ (*P*42/*mnm* S.G.), which exhibit different physical and chemical properties. According to the factor group analysis of the $D_{4h}^{19}$ symmetry, anatase TiO_2_ has six Raman-active modes (*A*_1g_ + 2*B*_1g_ + 3*E*_g_) [91], which appear at 144 (*E*_g_), 245 (*E*_g_), 326 (*B*_1g_), 403 (*B*_1g_), 516 (*A*_1g_ + *B*_1g_), and 639 cm^−1^ (*E*_g_) [92]. TiO_2_ nanoparticles have often been studied by Raman spectroscopy due to the unusual broadening and shifting of Raman bands as particle size decreases. Xu et al. attempted to explain these Raman band changes using a phonon confinement model [93]. Conversely, Choi et al. [94]. attributed the Raman shifts to the effects of particle size reduction on the force constants and vibrational amplitudes of the first-neighbor bonds. The Raman spectra of polycrystalline Li_1+x_Ti_2−x_O_4_ powders recorded with the 514.5 nm radiation are presented in Figure 14c, showing the end members (1) *x* = 1/3 (Li_4/3_Ti_5/3_O_4_) and (6) *x* = 0 (LiTi_2_O_4_) [95]. For LiTi_2_O_4_, the Raman spectrum shows five peaks, centered at 200, 339, 429, 494, and 628 cm^−1^ corresponding to the five Raman-allowed *A*_lg_ + *E*_g_ + 3*F*_2g_ modes of the spinel structure. In the range of 0.1 < x < 0.2, Raman spectra can barely be resolved. The absence of well-defined peaks for intermediate values of x provides evidence of the chemical inhomogeneity of Li_1+x_Ti_2−x_O_4_ due to a spinodal decomposition into a Li-poor phase approaching LiTiO_4_ and Li-rich phases approaching Li[Li_l/3_Ti_5/3_]O_4_. Raman spectra of pristine and chemically lithiated Li_4_Ti_5_O_12_ are shown in Figure 14d [96].

Rutile is also tetragonal with two TiO_2_ molecules per unit cell. There are four Raman-active modes with $D_{4h}^{14}$ symmetry of *A*_1g_ + *B*_1g_ + *B*_2g_ + *E*_g_. In a prior work, Narayanan observed eight rutile TiO_2_ Raman peaks at 150, 236 (*B*_1g_), 333 (IR, *A*_2u_), 300–440 (*E*_g_), 515, 589 (*B*_2g_), and 650 cm^−1^ using a mercury excitation source at λ = 546.1 nm [97]. The four Raman-active modes of rutile TiO_2_ were detected at 143 (*B*_1g_), 447 (*E*_g_), 612 (*A*_1g_), and 826 cm^−1^ (*B*_2g_) by Porto et al. [98]. Recently, Challagulla et al. reported characteristic stretching peaks of rutile TiO_2_ at 140, 430, and 590 cm^−1^ that correspond to the symmetries of *B*_1g_, *E*_g_, and *A*_1g_, respectively. In addition, another characteristic broad compound vibrational peak at 230 cm^−1^ arising from the multiple phonon-scattering processes was also clearly observed [99].

Spinel lithium titanate Li_4_Ti_5_O_12_ (LTO) has become the only mass-produced alternative to natural and artificial graphite as anode material for Li-ion batteries. The structural characterization of LTO by Raman and infrared spectroscopy was first reported by Proskuryakova et al. [100] and more recently by several groups of researchers [96,101,102,103]. The Raman spectrum of the LTO sample shows the five characteristic allowed lines at 234, 262, 342, 424, and 671 cm^−1^ that are very close to those previously reported. The five Raman-allowed phonon peaks are a feature of the spinel structure (*A*_1g_ + *E*_g_ + 3*F*_2u_). Other weak bands are related to multiple phonon-scattering processes. In particular, it was demonstrated that the frequencies of the Ti-O stretching vibrations lie in the range above 460 cm^−1^. The stretching–bending vibrations of the Li-O bonds in LiO_4_ and LiO_6_ polyhedra are recorded at 424 and 342 cm^−1^, respectively, while the peaks observed at frequencies below 300 cm^−1^ correspond to the bending modes of O-Ti-O (234 cm^−1^) and O-Li-O (160 cm^−1^) bonds. The Raman bands observed in the higher frequency range (671, 748 cm^−1^) were assigned to vibrations of Ti-O bonds in TiO_4_ tetrahedra. However, according to structural analyses, titanium atoms only occupy the 16*d* octahedral sites. Therefore, the high-frequency Raman bands at 671 and 748 cm^−1^ must be assigned to vibrations of Ti-O bonds in TiO_6_ octahedra (Figure 14a).

The β-phase Li_2_TiO_3_, which crystallizes in the monoclinic rock-salt structure (space group *C*2/*c*), can be described by the chemical formula Li_4/3_Ti_2/3_O_2_ or by the lamellar notation Li(Li_1/3_Ti_2/3_)O_2_. The Li and Ti cations occupy octahedral sites. The three non-equivalent positions of lithium, Li(1), Li(2), and Li(3), correspond to Wyckoff sites 8*f*, 4*d*, and 4*e*, respectively, while the two non-equivalent positions of titanium, Ti(1) and Ti(2), are located at the 4*e* sites. β-Li_2_TiO_3_ possesses a lamellar structure consisting of a stack of LiTi_2_ layers composed of Li(3) and Ti cations sharing the 4*e* position and lithium monolayers entirely occupied by Li(1) and Li(2) ions [104]. Of the 15 allowed Raman-active modes (7*A*_g_ + 8*B*_g_) with spectroscopic symmetry *C*_2h_^6^, only 11 bands are observed in the Raman spectrum of β-Li_2_TiO_3_. The high-wavenumber peaks, at 575 and 668 cm^−1^, are attributed to Ti-O stretching modes of TiO_6_ octahedra. The lattice modes, namely the *A*_g_(T) translational lattice mode, and the O-Ti-O bending modes appear at frequencies ν < 320 cm^−1^. The low-frequency peak at 98 cm^−1^ (of very weak intensity) is due to the *A*_g_(T) lattice mode. In the Li_2_TiO_3_ lattice, the lithium occupies both octahedral and tetrahedral positions. Consequently, the Li-O stretching modes of LiO_4_ and LiO_6_ are recorded at 358 and 430 cm^−1^, respectively.

The FTIR spectra of Li_4_Ti_5_O_12_ and Li_2_TiO_3_ nanopowders are shown in Figure 14b. The FTIR spectrum of Li_2_TiO_3_ displays nine IR bands. The broad peaks at 510 and 619 cm^−1^ are attributed to the antisymmetric stretching modes of Ti-O bonds. The low-frequency bands in the 350–450 cm^−1^ range result from a mixing of the O-Li-O and O-Ti-O bending modes, due to the occurrence of Li ions in the LiTi_2_ slabs. The band at 263 cm^−1^ is assigned to the stretching mode of Li-O bonds in LiO_6_ octahedra. The shoulder of the higher intensity peak at 642 cm^−1^ is thought to be related to local distortion of the crystal lattice, leading to a decrease in local structural symmetry and generating additional infrared bands [105]. The FTIR spectrum of Li_4_Ti_5_O_12_ is dominated by two strong absorption bands located at 653 and 504 cm^−1^ that are attributed to the asymmetric stretching modes (*F*_1u_ species) of TiO_6_^8−^ molecular units. It is particularly interesting to mention that the FTIR spectrum exhibits additional bands compared to factor group theory. For example, the doublet resolved at 653 (strong), 596 (weak), and 504, 455 cm^−1^ (shoulders) is assigned to the random distribution of Li^+^ ions in the octahedral 16*d* sites and the simultaneous occupation of these octahedral positions by Li and Ti ions of different charges and masses. Thus, these structural characteristics induce the IR activity of additional *F*_1u_ modes [106].

The Raman spectrum of LiTi_2_O_4_—is a superconductor (*T*_c_ ≈ 13 K)—displays the frequencies of the phonon modes at 200, 339, 429, 494, and 628 cm^−1^, corresponding to the five active Raman modes expected at the center of the Brillouin zone for a spinel-like structure (see Figure 14c) [95]. Li_5.9_Ti_5_O_12_ also exhibits five principal lines at 236, 352, 412, 494, and 674 cm^−1^. In contrast, the frequencies reported for the spinel LiTi_2_O_4_ are 200, 339, 429, 494, and 628 cm^−1^ (Figure 14d) [96]. The Raman spectra of chemically inserted Li_5.9_Ti_5_O_12_ show an overall broadening of the lines, and a new line is recorded at 494 cm^−1^, due to the coupling of modes involving differently bonded Li atoms in the structure of Li_5.9_Ti_5_O_12_.

Detailed analyses on the surface or subsurface defects of carbon-coated LTO were performed using Raman spectroscopy as a unique tool for the probing of structural defects. Pelegov et al. [107] investigated diluted defects in Li_4_Ti_5_O_12_ induced by carbon deposition, which have significant effects on the electrical and electrochemical properties. The band at 510 cm^−1^ and the shoulder at ~730 cm^−1^ were identified to correlate with the oxygen non-stoichiometry (V_O_ for short). Other Raman peaks located in the range below 200 cm^−1^ and at 400 and 620 cm^−1^ are supposed to have defect-caused origin, rather than that caused by the TiO_2_ presence. Recently, Nikiforov et al. [108] studied the ambiguity in the number of Raman bands of LTO through a low-temperature study and analysis of thermal shifts. They concluded that the approach with surplus bands is advantageous and recommend using major *F*_2g_ band shifts to estimate the sample heating.

## 6. Raman and FTIR Analysis of Electrolytes

### 6.1. Liquid Electrolytes

**Figure 15:**
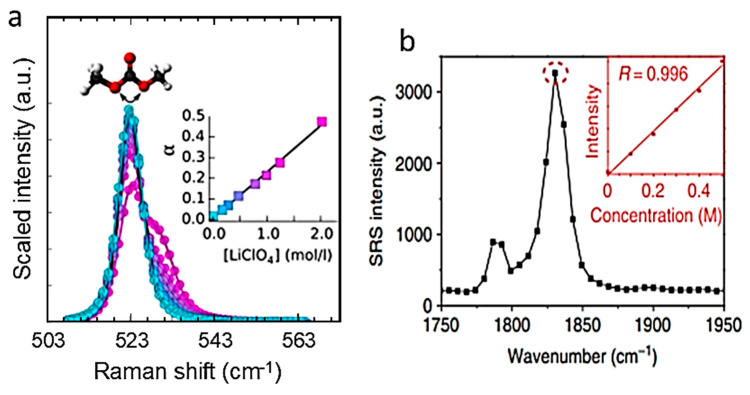
Raman microscopy of liquid electrolytes. (**a**) The Raman peak corresponding to the O-C-O deformation of DMC develops a sideband upon the addition of LiClO_4_. The curves are fits of the spectra using two Voigt profiles. Inset shows the fraction of intensity in the sideband, α, increases linearly with increasing LiClO_4_ concentration. The black line is a linear fit to the data. Reproduced from [110]. Copyright 2014 American Chemical Society. (**b**) The SRS spectrum of 0.5 mol L^−1^ LiBOB in TEGDME/PVdF-HFP gel electrolyte. The inset shows the linear concentration dependence between the Raman intensity at 1830 cm^−1^ and the LiBOB concentration from 0 to 0.5 mol L^−1^. Reproduced from [120] under a Creative Commons Attribution (CC-BY) license.

FTIR and Raman microscopy have been widely used as a nonperturbative tool to measure spatially resolved lithium-ion concentrations in liquid electrolytes. For an introduction to this subject, the reader can find preliminary overviews from Refs. [109,110,111,112,113,114]. Considering the benefit of a spatial resolution lower than 1 µm in the lateral direction and 8 µm in the axial direction of confocal Raman microscopy, Forster and co-workers [115] combined this high-spatial-resolution technique with a simple microfluidic device, enabling measurement of the diffusion coefficient of lithium ions (LiClO_4_) in dimethyl carbonate (DMC) in two different concentration regimes. They consider the Raman peak corresponding to the O-C-O deformation of DMC at 523 cm^−1^, which develops a sideband upon the addition of LiClO_4_ (Figure 15a). As Raman spectroscopy can detect [Li^+^] by Li^+^-solvent interactions [115,116,117] or anion concentration based on “electroneutrality” [117], this technique has been widely employed to study common Li salts (e.g., LiClO_4_ [118,119], LiBOB [120], and LiTFSI [121]), and solvents (e.g., DMC [118] and tetraglyme [122]). Cheng et al. [120] reported the linear concentration dependence between the Raman intensity of the BOB^−^ vibration at 1830 cm^−1^ (Figure 15b) and the LiBOB concentration from 0 to 0.5 mol L^−1^ in tetraethylene glycol dimethyl ether (TEGDME) with 22 wt.% poly (vinylidene fluoride-*co*-hexafluoropropylene) (PVdF-HFP) as gel electrolyte. The most popular salt in LIBs, LiPF_6_, has been studied using Raman for structural analysis. LiPF_6_ has been shown to induce a fluorescence background in Raman spectroscopy experiments with its strong PF_6_ Raman-active band.

**Figure 16:**
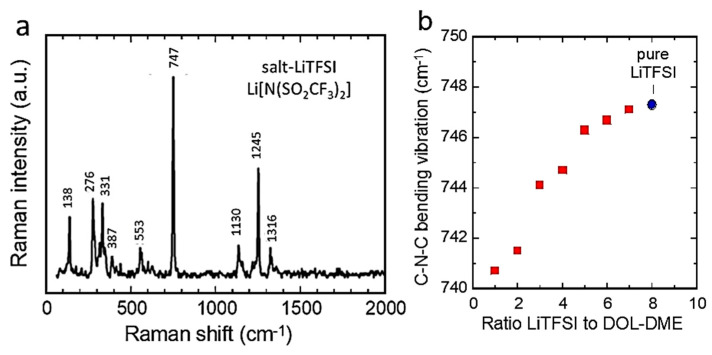

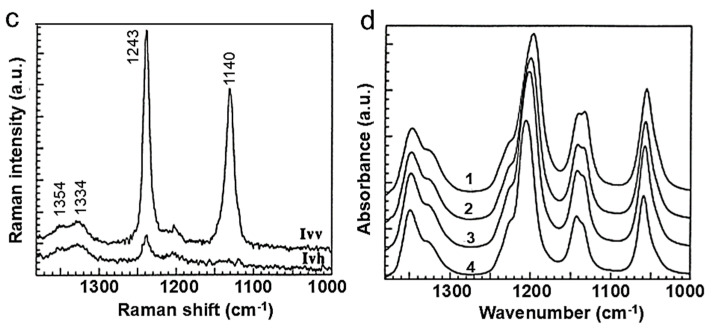
(**a**) FT-Raman spectrum of pure LiTFSI. (**b**) Frequency shift in the C-N-C bending vibration with different ratios of LiTFSI to DOL-DME (1:1 by volume). Reproduced from [118]. Copyright 1989 Elsevier. (**c**) Room-temperature Raman spectra at two different polarizations of (H_2_O)_266_/LiTFSI. (**d**) Infrared spectra at 25 °C of aqueous solutions of mole fraction [LiTFSI]/[H_2_O] = 1/266 (1), 1/50 (2), 1/30 (3), and 1/20 (4). The intensity of the IR spectra 1, 2, and 3 has been multiplied, respectively, by 5.5, 2.0, and 1.4 for a convenient comparison. Reproduced with permission from [124]. Copyright 1998 Elsevier.

Suo et al. [123] investigated Li^+^-ion transport in solvent-in-salt (SIS) electrolytes using Fourier transform Raman (FT-Raman) spectroscopy. The spectra produced at different ratios of salt to solvent are compared to establish a relationship between salt concentration and lithium-ion transport properties. The lithium bis(trifluoromethanesulphonyl)imide (LiN(SO_2_CF_3_)_2_, LiTFSI) salt and 1,2-dimethoxyethane (DME) and 1,3-dioxolane (DOL) (1:1) by volume were mixed by a ratio of mole number to volume. The Raman pattern of LiTFSI recorded at 1064 nm with an InGaAs laser displays an intense Raman peak at 747 cm^−1^ (Figure 16a), which belongs to the bending vibration of the C-N-C group. The variation in the C-N-C bending frequency as a function of the LiTFSI/DOL-DME ratio is presented in Figure 16b. Four types of ion pairs were defined by different C-N-C bending vibration positions: free ion (736–738 cm^−1^), loose ion pair (740–742 cm^−1^), intimate ion pair (744–746 cm^−1^), and aggregated ion pair (746.5–747.3 cm^−1^). Meyer et al. [109] reported the operando measurements of electrolyte Li-ion concentration during fast charging with the FTIR/ATR technique. This study focused on the shift in infrared absorption bands in the solvent by solvation in the presence of lithium ions. The infrared absorption bands shifted by Li salt and the unshifted bands of ethyl methyl carbonate (EMC) and ethylene carbonate (EC) were compared to the ion concentration changes induced during charge/discharge cycles. Lithium concentrations were calibrated using EC/EMC/LiPF_6_ electrolytes with known lithium contents in a graphite-anode Li-ion half-cell. The parasitic reactions between NMC electrodes and a carbonate electrolyte were also identified using in situ ATR-FTIR spectroscopy.

The characteristic vibrations of the TFSI anion and P(EO)_15_/LiTFSI complexes were compared using spectral features of aqueous solutions to estimate the conformational isomerism and ionic pairing effects [124]. The Raman and infrared spectra are presented in Figure 16c and 16d, respectively. The broad, structured envelope located in the range 1300–1380 cm^−1^ is attributed to *ν*_a_(SO_2_), while at least two intense main components are observed in IR and two weak, non-polarized components are visible in Raman (Table 3). The symmetric stretching mode *ν*_s_(SO_2_) is also known to appear in a relatively narrow frequency range, for example, at 1160 ± 30 cm^−1^ for R-SO_2_R′ compounds and at 1163 cm^−1^ for dimethylsulfamyl chloride (CH_3_SO_2_)_2_NH. The line observed at 1131 cm^−1^ in aqueous solution and at 1140 cm^−1^ in PEO appear as intense, polarized Raman lines (Table 3). The corresponding IR absorption band is in the form of a doublet at 1143–1135 cm^−1^, even in diluted solution.

Zhang et al. [125] employed ATR-FTIR spectroscopy to investigate, at a microscopic level, the oxidation reaction of crystalline NMC811 electrodes in various electrolytes. They identify the species resulting from the dehydrogenation of EC, vinylene carbonate (VC), and oligomers containing EC-type rings when charging the NMC811 electrode up to 3.8 V vs. Li^+^/Li. Recently, Fawdon et al. [126] monitored the evolution of electrolyte concentration gradients over time using operando Raman microspectroscopy combined with potentiostatic electrochemical impedance spectroscopy. This integrated approach enabled the quantification of the Fickian “apparent” diffusion coefficient, the cation transference number, and the thermodynamic factor within a single experimental setup. Lithium bis(fluorosulfonyl)imide (LiFSI) dissolved in tetraethylene glycol dimethyl ether (tetraglyme, G4) was employed as a model electrolyte system.

### 6.2. Solid-State Electrolytes (SSEs)

Inorganic oxide and sulfide materials have recently been studied as solid electrolytes as fast ionic conductors (FICs) for all-solid-state batteries (ASSBs) owing to their high safety profile, wide temperature window, and better mechanical properties than those of liquid electrolytes [127]. Vibrational spectroscopies were widely used to characterize the structure of FICs, local structure change during the crystallization, interfacial evolution, cationic impurity concentration, etc. For example, in an early and influential study, Börjesson and Torell employed Raman scattering to investigate the fast-ion-conducting phases of Li_2_SO_4_ and LiAgSO_4_, comparing their spectra with those of the poorly conducting analogue Na_2_SO_4_. Their results indicated that SO_4_^2−^ reorientations are characteristic features of fast-ion-conducting sulfates, consistent with prior observations from neutron-scattering experiments. The linewidths associated with these orientational modes were found to follow an Arrhenius dependence, yielding activation energies of 0.40 and 0.72 eV [128].

#### 6.2.1. Sulfide Electrolytes

**Figure 17:**
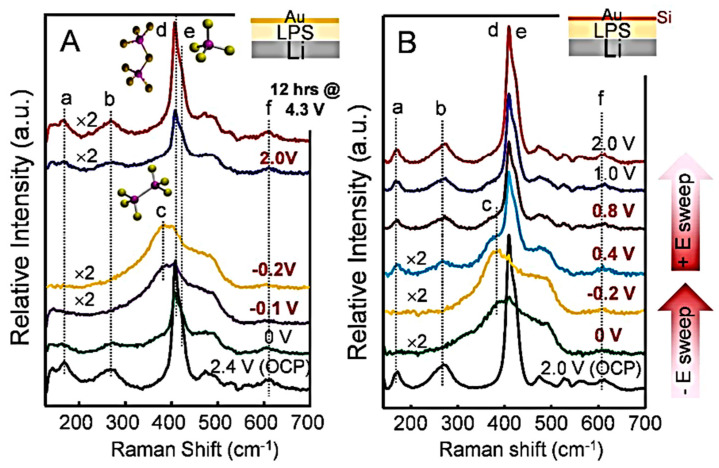
In situ Raman spectra obtained at the Au electrode in (**A**) Li/LPS/Au cell and (**B**) Li/LPS/SiAu cell. Spectra were collected with a 532 nm laser as the excitation source. Each spectrum was a co-addition of 60 spectra, each with a 2 s integration time. Six LPS signature peaks are observed (a) lattice deformation, (b) δ_def_(S−P−S) in PS_4_^3−^, (c) νs(PS_3_) and ν(P−P) in P_2_S_6_^4−^, (d) νs(PS4) in P_2_S_7_^4−^, (e) ν_s_(PS_4_^3−^), (f) ν_as_(PS_4_^3−^). Reproduced with permission from [131]. Copyright 2018 American Chemical Society.

Thiophosphate-based SSEs, such as Li_3_PS_4_, Li_7_P_3_S_11_, Li_4_P_2_S_7_, and Li_10_GeP_2_S_12_, are superionic conductors, which exhibit Li^+^ conductivities comparable to liquid electrolytes. Zhang et al. [129] reported the inter- and intra-cycle interfacial evolution of a LiNi_0.8_Co_0.1_Mn_0.1_O_2_ (NMC811)|Li_6_PS_5_Cl|Li cell. Argyrodite materials Li_6_PS_5_*X* (*X* = Cl, Br, I) consist of PS_4_ tetrahedra, all linked by interstitial site around halide ions, resulting in the ionic formula (Li^+^)_6_(PS_4_^3−^)S^2−^X^−^. During CV cycling, the Raman peaks exhibit a fluctuating variation at 418 and 425 cm^−1^. The peak located at 423 cm^−1^ is associated with the stretching mode of ortho-thiophosphate PS_4_^3−^ with the ionic formula (Li^+^)_6_(PS_4_^3−^)S^2−^Cl^−^. The other peak at 418 cm^−1^ originates from different PS_4_^3−^ vibrational modes due to the presence of another anion. Unlike many reports on sulfide-type electrolytes [130], there is no obvious molecular conversion of PS_4_^3−^ to P_2_S_6_^4−^ (at 410 cm^−1^) and P_2_S_7_^4−^ (at 390 cm^−1^), which suggests that the composition and structure of Li_6_PS_5_Cl at the interface remain intact during the first cycling. Sang et al. [131] evaluated the effect of two interlayer materials using lithium thiophosphate Li_7_P_3_S_11_ (LPS) as SSE. Figure 17A shows a series of Raman spectra measured at the LPS/Au interface at various potentials. Six LPS signature peaks are observed. The main peak (d) located at 411 cm^−1^ and the shoulder (e) at 426 cm^−1^ are associated with the symmetric stretching mode of PS_4_ in P_2_S_7_^4−^ and PS_4_^3−^, respectively. The peak (a) observed at 178 cm^−1^ corresponds to the lattice deformation mode, and the peak (b) at 272 cm^−1^ is assigned to the δ_def_(S-P-S) deformation mode of PS_4_^3−^. The peak (f) at 610 cm^−1^ arises from the asymmetric stretching mode of PS_4_^3−^. The peak (c) at 380 cm^−1^ is attributed to the ν_s_(PS_3_) and ν(P-P) vibrations in P_2_S_6_^4−^. Figure 17B presents the in situ Raman spectra recorded at the LPS/SiAu interface as a function of potential. It is observed that the Raman features of the pristine SiAu-coated LPS surface are similar to those of the Au-coated surface. The intensity of peak (c) associated with the vibration of P_2_S_6_^4−^ increases, while peaks of PS_4_^3−^/P_2_S_7_^4−^ (a, b, and d, f) decrease at 0 V and −0.2 V [131].

**Figure 18:**
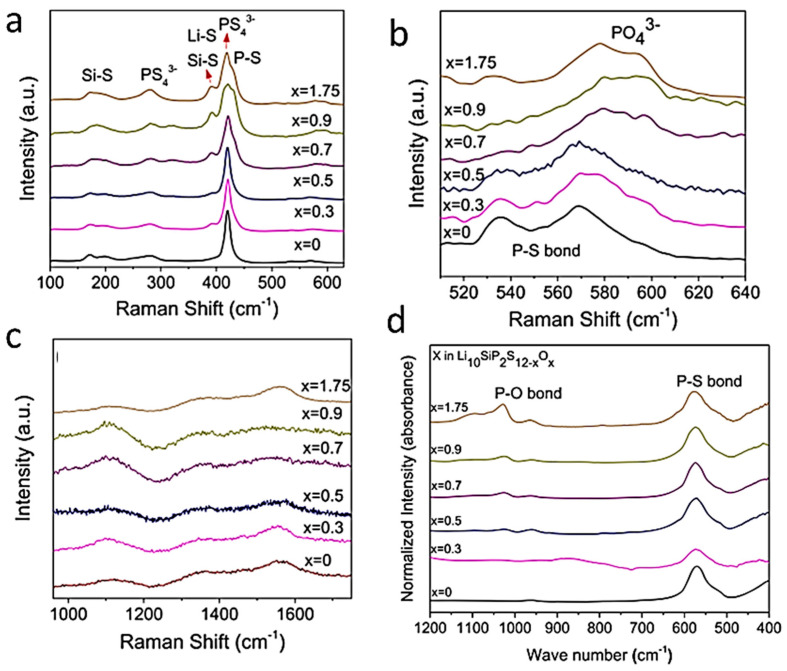
Raman spectra of LSPSO solid electrolytes in three spectral regions: (**a**) 100−500, (**b**) 500−650, and (**c**) 1000 to 1800 cm^−1^. (**d**) Mid-infrared spectra of LSPSO from 400 to 1200 cm^−1^ showing the growth of the vibration peak of the P-O bonds. Reproduced with permission from [135]. Copyright 2019 American Chemical Society.

Dietrich et al. [132] investigated the crystal structure of Li_2_S-P_2_S_5_ glass electrolytes using multiple analytical techniques, including Raman spectroscopy performed with a 532 nm excitation wavelength and a laser power of 0.2 mW. Quantitative peak-deconvolution results obtained from Raman and ^31^P MAS NMR spectroscopy were reported for different stoichiometries (Li_2_S)_x_(P_2_S_5_)_100−x_. Raman spectra were deconvoluted in the 350–450 cm^−1^ region using least-squares fitting with Gaussian–Lorentzian product functions (20:80). The analysis revealed a continuous decrease in the fraction of di-tetrahedral P_2_S_7_^4−^ anions with increasing Li_2_S content, accompanied by a corresponding increase in the proportion of mono-tetrahedral PS_4_^3−^ units. At high Li_2_S content, PS_4_^3−^ monomers are mainly found, while at lower Li_2_S content, P_2_S_7_^4−^ dimers are the dominant species. Over the full compositional range, P_2_S_6_^4−^ dimers can be found with an amount of 10 to 15 at% P. At a low fraction of Li_2_S of 60 mol%, PS_3_ chains are observed. Preefer et al. [133] reported a rapid microwave-assisted synthesis of 70Li_2_S-30P_2_S_5_ and Li_7_P_3_S_11_ material. The composition and the appropriate ratios of isolated and corner-sharing tetrahedra in these semicrystalline systems were characterized by Raman spectroscopy in the region extending from 350 to 450 cm^−1^. The glass samples show no signatures of Li_4_P_2_S_6_, and the glass–ceramic samples show a hint of the P_2_S_6_^4−^ dumbbell centered at 390 cm^−1^. Li et al. [134] studied the reactions at the interface between LiNi_0.80_Co_0.15_Al_0.05_O_2_ (NCA) and Li_10_GeP_2_S_12_ (LGPS) using in situ and ex situ Raman spectroscopy (using excitation laser line of 532 nm and resolution of ≤0.7 cm^−1^) and probing the characteristic peaks at 181, 287, 373, 433, and 1559 cm^−1^. A new peak at 1441 cm^−1^ appears in the NCA/LGPS mixture layer after staying for 60 min. Moreover, this peak intensity strengthens gradually with time, meaning that chemical side reactions between NCA005 and LGPS occur once they are in contact. The peaks located below 400 cm^−1^ are also modified. These spectroscopic features are well correlated with electrochemical spectroscopy (EIS) results, showing the increase in the cathode/solid electrolyte interfacial impedance upon cycling in the Nyquist plots. Li_10_SiP_2_S_12_ (LSPS), which has an Li_10_GeP_2_S_12_ (LGPS)-type crystalline structure, was synthesized by solid-state reaction and then doped with oxygen to produce oxysulfide compositions of the general formula Li_10_SiP_2_S_12−x_O_x_ (LSPSO), with 0 ≤ *x* ≤ 1.75 [135]. By means of Raman and FTIR spectroscopy, the effect of O-doping on the crystal structure of LSPSO was analyzed (see Figure 18). The Raman spectra (Figure 18a–c) show three regions of interest. In the spectral region 100−500 cm^−1^ (Figure 18a), the peaks at 172 and 197 cm^−1^ are assigned to the S-bond bending modes of SiS_4_ tetrahedra, whereas the peak at 280 cm^−1^ arises from the bending modes of PS_4_ tetrahedra. The peak at 390 cm^−1^ corresponds to the stretching mode of SiS_4_ polyhedra. In the spectral region 500−650 cm^−1^, two new peaks appear upon O doping, which are due to the formation of oxy-sulfide PS_4−z_O_z_ units (Figure 18b). In the spectral region 800–1400 cm^−1^ (Figure 18c), the weakest intensity peaks are attributed to the pure oxide polyhedral vibrations. Mid-infrared spectra, around 966 and 1027 cm^−1^, show a clear increase in the intensity of peaks attributed to the O bond, corresponding to the vibrations of the Li_3_PO_4_ crystal and its tetrahedral PO_4_ unit (Figure 18d). A single broad peak, due to the P-S bond, was observed around 574 cm^−1^.

Chen et al. investigated the chemical evolution of the sulfide-based solid electrolyte Li_6_PS_5_Cl (LPSCl) during air exposure in order to assess its compatibility with dry-room processing environments [136]. Changes in the chemical bonding were monitored using both Raman and FTIR spectroscopy. FTIR spectra revealed strong O-H stretching bands between 3000 and 3500 cm^−1^, indicating extensive hydrolysis upon exposure to moisture. The appearance of carbonate-related features—namely the asymmetric stretching at 1427 cm^−1^ and the out-of-plane bending at 867 cm^−1^—further suggested reactions with atmospheric CO_2_ under ambient conditions. In the pristine state, LPSCl exhibits a characteristic PS_4_^3−^ stretching band near 425 cm^−1^ in the Raman spectrum (532 nm excitation). Combined with ^31^P and ^7^Li NMR analyses, the spectroscopic results demonstrate that LPSCl is highly susceptible to chemical degradation upon moisture exposure.

#### 6.2.2. Oxyhalide-Type Solid Electrolytes

Halide-type and oxyhalide solid electrolytes (Li_3x_TaO_3x_Cl_5−3x_, 0.8/3 ≤ x ≤ 1.4/3) have recently been developed as promising materials exhibiting enhanced ionic conductivities (up to 9 mS cm^−1^ at 30 °C). The local structural environments and Li^+^ transport pathways within the Li_3x_TaO_3x_Cl_5−3x_ series were elucidated using a combination of Raman spectroscopy, X-ray photoelectron spectroscopy (XPS), and X-ray absorption spectroscopy (XAS). The R3c structure of LiTaO_3_ was confirmed, and characteristic vibrational modes were identified, including high-frequency Ta-O stretching vibrations, O-Ta-O bending modes below 450 cm^−1^, and Li-O stretching modes below 400 cm^−1^. Li_1_._2_TaO_1_._2_Cl_3_._8_ exhibits distinct Raman signatures, including new bands at 212 and 409 cm^−1^ that correspond to Ta-Cl vibrations within a trigonal bipyramidal TaCl_5_ unit. This observation suggests a structural transition from Ta_2_Cl_10_ dimers to TaCl_5_ trigonal bipyramidal monomers during mechanochemical synthesis of LiTaO_3_, TaCl_5_, and LiCl [137].

Kwak et al. reported Raman and XAFS analyses of Fe^3+^-substituted Li_2_ZrCl_6_ solid electrolytes prepared by ball milling (BM) and heat treatment at 260 °C (HT). Under 514.5 nm excitation, both BM- and HT-Li_2_ZrCl_6_ samples display two intense Raman peaks at ~325 and ~161 cm^−1^, assigned to the A_1_g stretching and F_2_g bending modes typical of the elpasolite family [138]. Song et al. investigated the structural properties of amorphous oxyhalide electrolytes, Li_4*x*_La*_x_*Ta_1−*x*_O_2*x*_Cl_5−2*x*_ (*x* = 0.25, 0.35, and 0.5), using XRD, SEM, XPS, and Raman spectroscopy to identify local environments influencing ionic transport [139]. Li_2_ZrCl_6_ and 5 mol% Mn-doped Li_2_ZrCl_6_ were further analyzed using powder XRD, ^7^Li NMR, XPS, and Raman spectroscopy (100–550 cm^−1^, 532 nm, and 8 mW). Pristine Li_2_ZrCl_6_ exhibits peaks at 165 and 326 cm^−1^, while increasing Mn content leads to peak broadening, attributable to reduced crystallinity in Mn-doped samples [140].

Complementary spectroscopic techniques—including Raman and FTIR—have proven essential for studying air sensitivity and degradation pathways of halide solid electrolytes [141,142,143,144]. The influence of atmospheric exposure on Li_3_InCl_6_ was first clarified through operando optical microscopy and Raman spectroscopy. As exposure time increases (30% relative humidity), characteristic peaks at 159 and 184 cm^−1^ (In-Cl in-plane and out-of-plane bending) and 280 cm^−1^ (In-Cl symmetric stretching) exhibit decreased intensity and increased broadening, while a new peak at ~131 cm^−1^ emerges, attributed to In_2_O_3_ formation [142]. A subsequent study by the same group assessed humidity tolerance in Li_3_Y_1−x_In_x_Cl_6_ electrolytes. For Li_3_YCl_6_, exposure induces broad Raman features corresponding to Y^3+^-H_2_O-Cl^−^ and Y-Cl vibrations, whereas In-rich compositions display markedly different spectra, including a characteristic 282 cm^−1^ mode [144]. Bilo et al. combined XAS and Raman spectroscopy to investigate local coordination changes in pristine Li_3_InCl_6_ exposed to ~60% relative humidity. The Raman spectrum of air-exposed samples becomes similar to that of LiCl, indicating partial decomposition of Li_3_InCl_6_ [143]. Usami and co-workers further studied moisture effects in Li_3_YCl_6_ and Li_3_InCl_6_ using ATR-FTIR. Li_3_YCl_6_ presents broad OH-derived features, whereas Li_3_InCl_6_ exhibits sharper peaks, suggesting that H_2_O molecules are more strongly immobilized in the latter [144]. Li and co-workers applied a multimodal operando approach—combining optical microscopy, Raman spectroscopy, synchrotron-based X-ray powder diffraction, and in situ X-ray absorption near-edge structure (XANES)—to monitor the degradation of Li_3_InCl_6_ upon exposure to air. Raman measurements were conducted using a 532.4 nm (≈2.3 eV) laser, providing chemical information from the surface region. Complementary Cl K-edge and In L_3_-edge XANES spectra were collected using synchrotron X-rays with photon energies of several keV, enabling bulk-sensitive probing depths of several micrometers. Because air-exposed Li_3_InCl_6_ does not exhibit distinct XRD reflections, the evolution of Raman and XANES signatures was used to identify decomposition pathways. Spectral features corresponding to In-containing secondary phases—such as InOCl or Li_3_InCl_6−x_(OH)_x_—were detected, and the formation of In_2_O_3_ was identified as the final degradation product [145].

#### 6.2.3. Oxide Glasses

**Figure 19:**
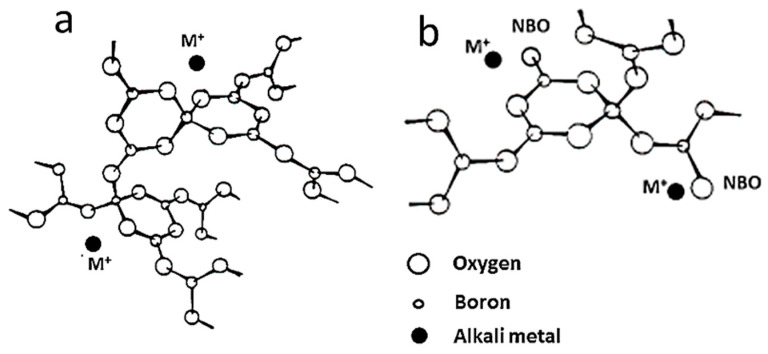
Alkali borate glass networks formed (**a**) at low and (**b**) at high alkali oxide concentrations. M^+^ is the alkali metal, and NBO is the non-bridging oxygen.

Raman and infrared spectroscopies are the most effective analytical methods for studying the structure of highly disordered materials such as amorphous materials, glasses, and all materials without long-range order. These techniques give typical spectral patterns corresponding to constituent functional groups of the frames and lead to the identification of the crystalline organization and the diagnosis of intermolecular interactions. By probing the short-range order of materials, they have a key impact for investigating fast ion-conducting glasses (FIC glasses) when diffraction techniques are ineffective. The adoption of vibrational spectroscopies allows highly sensitive estimation of crystallinity and a non-destructive means to study the chemical composition. FIC borate glasses containing lithium-ion carriers have been widely investigated in the ternary system B_2_O_3_-*x*LiO-*y*Li_n_*X*, where *X* represents halide anions. In such solid-state electrolytes, the ionic conductivity is all the more important as the content of modifying oxide (Li_2_O) and lithium “doping salt” (Li_n_*X*) is higher. The anion *X* is usually a halogen (*n* = l), ${SO}_{4}^{2-}$ (*n* = 2), etc. [146]. Krogh-Moe [147] determined the structure of the glassy compound B_2_O_3,_ consisting of a random framework of boroxol rings and BO_3_ triangles connected by a bridging oxygen atom bond of type B-O-B. The addition of alkali oxides modifies the boroxol rings and forms complex borate groups with one or two tetra-coordinated boron atoms. Figure 19 illustrates alkali borate glass networks formed at low and high concentrations of alkali oxide.

**Figure 20:**
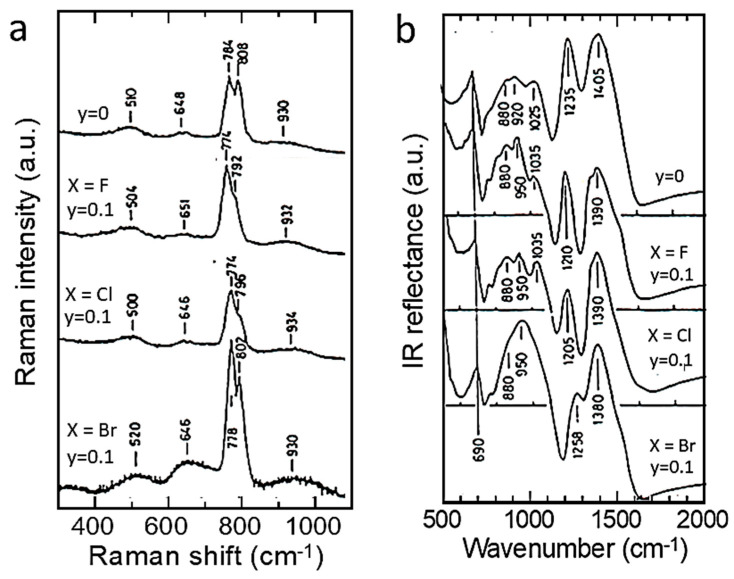
(**a**) Raman spectra and (**b**) mid-infrared reflectance spectra of lithium halogenoborate glasses B_2_O_3_-0.2LiO-*y*Li*X* (*X* = F, Cl, Br, and I). Reproduced from [150]. Copyright 1989 Elsevier.

The implementation of Raman scattering and infrared spectroscopies combined with data analysis has assumed a better understanding of lithium borate glasses, providing not only their structural properties as a function of the nature and content of doping salts [148,149,150,151,152,153,154,155,156,157], but also demonstrating the conduction mechanism by studying the charge carrier dynamics [151]. For example, spectroscopic data from Raman scattering and infrared reflectance have shown that the factor affecting the fraction of 4-coordinated boron atoms in *x*Li_2_O-*y*LiCl-B_2_O_3_ ternary glasses is related to the O/B atomic ratio, and that the halide ion indeed causes major lattice modifications [150]. The spectroscopic investigations of lithium borate glasses B_2_O_3_-Li_2_O doped with various lithium salts Li*X* (where *X* = F, Cl, Br, or I) show local structural changes attributed to interactions between the vitreous network and the doping salt anion (see Figure 20). In the case of a particular halide anion in ternary glasses, lattice modifications are evidenced by the appearance of a shoulder at approximately 720 cm^−1^ in the Raman scattering spectra. The frequency of this additional feature is shifted towards low energies, and its intensity increases with the doping salt concentration. The bandwidth of this peak is narrow compared to that of the other bands in the glass spectrum. At the same time, the frequency of the band at 520 cm^−1^ increases slightly. This band is attributed to the in-plane movement of the bridging oxygen and the boron, with a little or no displacement of this atom. Moreover, the Raman bands corresponding to the vibrations of groups containing non-bridging oxygen (NBO) atoms located at 960 and 1480 cm^−1^ have a stronger intensity. This point is very interesting, because it indicates the presence of groups with NBO atoms such as metaborate or ditriborate groups [150].

**Figure 21:**
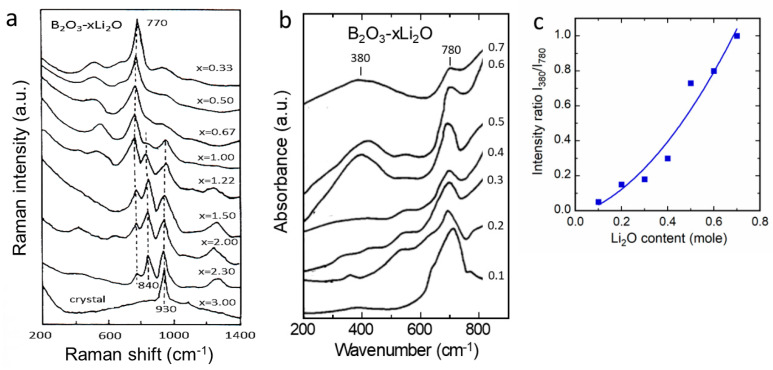
(**a**) Raman spectra of B_2_O_3_-*x*Li_2_O (0.33 ≤ *x* ≤ 2.30) glasses with high lithium oxide content. The spectrum of Li_3_BO_3_ crystal is given as standard. (**b**) Infrared absorption spectra with 0.1 ≤ *x* ≤ 0.7 in the range 200–800 cm^−1^. (**c**) Intensity ratio of the absorption peak at 380 and 780 cm^−1^ as a function of the Li_2_O content. Reproduced with permission from [150]. Copyright 1989 Elsevier.

The vibrational features of the binary B_2_O_3_-*x*LiO glassy electrolytes are illustrated in Figure 21 [150]. As shown in Figure 21a, the Raman spectra of B_2_O_3_-*x*LiO glasses show a significant transformation upon increasing the concentration of the glass modifier Li_2_O. Upon addition of Li_2_O in the glass framework, one observes the vanishing of the very intense and strongly polarized narrow Raman peak at ca. 806 cm^−1^, which is a characteristic of boroxol rings (at *x* ≈ 0.3), and the appearance of a new band at 780 cm^−1^ corresponding to the trigonal deformation of six-membered borate rings with one or two BO_4_ units. However, the addition of Li_2_O oxide leads to an increase in intensity of the peaks near 840 and 930 cm^−1^ and a decrease in the intensity of the peak at 770 cm^−1^. At the end, the peak at 930 cm^−1^ becomes the most intense in the glass with the highest lithium oxide content. Such features are also observed in the Li_3_BO_3_ crystal. The peak located at 840 cm^−1^ is the strongest pattern for the B_2_O_3_-2Li_2_O glasses, corresponding to the vibrational mode of lithium pyroborate Li_4_B_2_O_5_. The strong band centered at 770 cm^−1^ observed for relatively low Li_2_O content is assigned to borate rings with one or two BO_4_ units. On the other hand, the bands near 840 and 930 cm^−1^ are attributed to vibrational modes of the pyroborate B_2_$O_{5}^{4-}$ and orthoborate B$O_{3}^{3-}$ groups, respectively. As a general rule, the intensity of the Raman bands due to the vibrations of borate groups decreases with the increase in the Li_2_O concentration in the highly modified glasses. When the Li_2_O concentration exceeds 50 mol.% (*x* = 1), the main Raman-active structural entities are the pyroborate and orthoborate groups. For the composition B_2_O_3_-2.33Li_2_O, the glass structure is not built by a covalent network but by discrete borate anions such as pyroborate and orthoborate groups. The FTIR absorption spectra (in the far-infrared region, 200–800 cm^−1^) of the binary glasses B_2_O_3_-*x*Li_2_O glasses with 0.1 ≤ *x* ≤ 0.7 are displayed in Figure 21b. Two main absorption bands are located at 380 and 700 cm^−1^. The band at 700 cm^−1^ is attributed to the bending vibration of the B-O-B link in the boron–oxygen network, while the 380 cm^−1^ band is assigned to the vibrational motion of Li ions. Figure 21c illustrates the variation in the *I*_380_/*I*_780_ ratio against Li_2_O concentration. Its continuous increase with Li_2_O content reflects the localized interaction of the cations with the anionic sites, i.e., BO_4_ units of the network [150].

**Figure 22:**
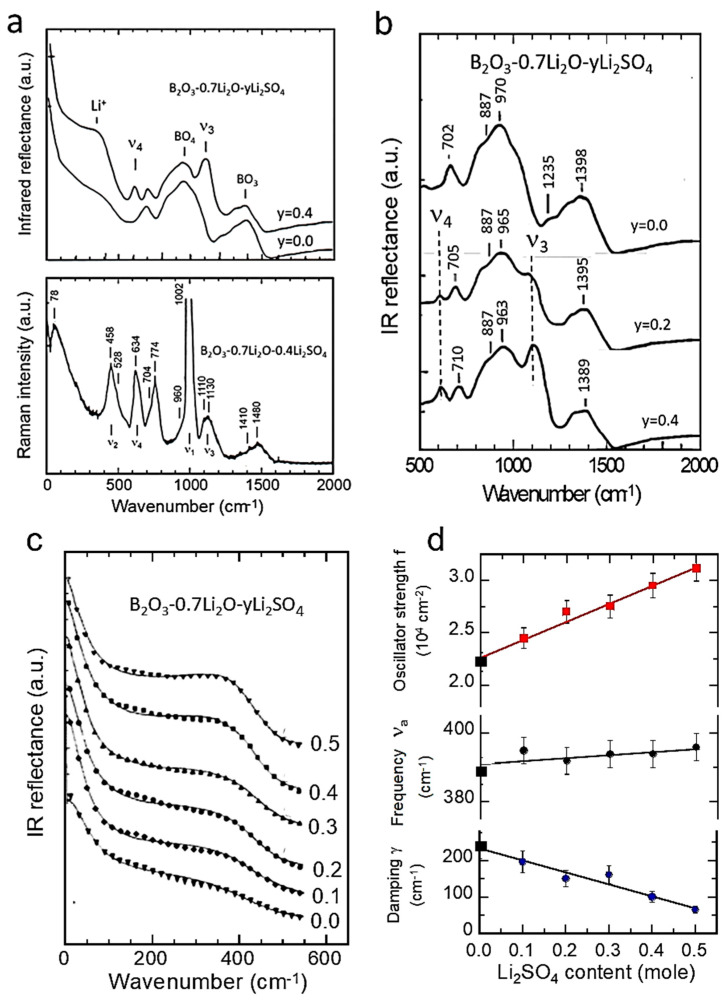
(**a**) Raman and FTIR reflectance spectra of B_2_O_3_-0.7Li_2_O-0.4Li_2_SO_4_ glasses. In the Raman spectrum, the “boson peak” is indicated at 80 cm^−1^, whereas the cation motion band is viewed at 370 cm^−1^ in the infrared spectrum. The binary glass B_2_O_3_-0.7Li_2_O is shown as standard. (**b**) Mid-infrared reflectance spectra of the B_2_O_3_-0.7Li_2_O-*y*Li_2_SO_4_ glasses (*y* = 0.0, 0.2, and 0.4). ν_3_ and ν_4_ are the spectral fingerprints of the sulfate dopant. Reproduced from [151]. Copyright 1989 Elsevier. (**c**) Far-infrared spectra of B_2_O_3_-0.7Li_2_O-*y*Li_2_SO_4_ (0.0 ≤ *y* ≤ 0.5). (**d**) Variation in the oscillator strength *f*, attempt frequency ω_o_, and damping factor γ, as functions of the dopant concentration in borate glass. Reproduced from [157]. Copyright 1989 Elsevier.

Figure 22 shows the Raman and FTIR reflectance spectra of B_2_O_3_-0.7Li_2_O-yLi_2_SO_4_ ternary glasses. The low-frequency portion of the Raman spectrum of the B_2_O_3_-0.7Li_2_O-0.4Li_2_SO_4_ glass is dominated by the so-called boson peak (BP). This pattern is a universal low-energy excitation in highly disordered materials appearing in the frequency range of 10~100 cm^−1^. The BP located at ca. 80 cm^−1^ in the Raman spectrum of B_2_O_3_-0.7Li_2_O-0.4Li_2_SO_4_ results from the competition between the decreasing density of vibration states and the increasing thermal population, while the vibration frequencies decrease toward zero [151].

The Raman scattering spectra of ternary glasses show bands attributed to the internal vibrations of tetrahedral sulfate ions located at approximately 458, 634, 1002, and 1130 cm^−1^, which correspond to the fundamental vibrations ν_2_, ν_4_, ν_1_, and ν_3_ of SO_4_^2−^ ions, respectively [152,153,154,155,156,157]. At high Li_2_O content (*x* = 0.7) in B_2_O_3_-0.7Li_2_O-*y*Li_2_SO_4_ glass, the dissociation of the lithium sulfate into a boron–oxygen network was identified by spectroscopic experiments on diborate glasses doped with lithium or potassium sulfate. Analysis of the IR reflectivity spectrum displayed in Figure 22c reveals that the cation motion band is visible at 370 cm^−1^. Compared with the spectrum of undoped B_2_O_3_-0.7Li_2_O glass, some changes have occurred in the O/B network. In the infrared spectrum of binary glasses, the shape of the BO_4_ band, with its maximum centered at 970 cm^−1^, indicates that BO_4_ units belong mainly to the diborate groups. The presence of a small band centered at 1235 cm^−1^ highlights the presence of trigonal BO_3_ units with NBO atoms. As in the Raman spectra, new bands are observed in the infrared reflectivity spectra of ternary glasses. These bands, whose intensity increases with sulfate content, correspond to the ν_4_ and ν_3_ vibrational modes of tetrahedral SO_4_^2−^ ions.

Figure 22c displays the far-infrared spectra of B_2_O_3_-0.7Li_2_O-*y*Li_2_SO_4_ (0.0 ≤ *y* ≤ 0.5). They can be divided into two parts: the low frequencies, which concern the dynamics of charge carriers, and the high frequencies, which concern the host. Thus, infrared spectroscopy is a complementary method to infer the frequency-dependent conductivity at low frequencies and the dynamics of the conduction ion [152]. The dielectric function of any material is obtained from the infrared reflectance response, *R*(ω), through the Kramer–Krönig inversion:

(23) $$\varepsilon \left(\omega \right)={\left(\frac{1+\sqrt{R(\omega )}}{1-\sqrt{R(\omega )}}\right)}^{2}$$

Then, the frequency-dependent conductivity, σ(ω), in the band can be calculated from the following relationship:

(24) $$\varepsilon \left(\omega \right)=\varepsilon_{\infty }-\frac{4\pi \sigma \left(\omega \right)}{i\omega }$$

In these calculations, the adjustable parameters are the oscillator strength *f*, the attempt frequency of the Li motion is ν_a_, and the damping factor is γ (Figure 22d). In the case of the (100 − *x*)B_2_O_3_-*x*Li_2_O glasses, the frequency of vibration is ν_a_ = 404 cm^−1^ for *x* = 0.32. When the activated process involves a vibrationally excited ion moving to an adjacent site, the activation energy *E*_σ_ for ionic conduction can be deduced from the following relation [157]:

*E*_σ_ = ½ *µ*ν_a_^2^a_o_^2^ (25)

where *µ* represents the reduced mass of the mobile cation, and *a*_o_ is the distance between adjacent sites. By taking the frequency corresponding to the absorption maximum, ν_a_, of the complete far-infrared envelope as the representative of attempted cation frequency, Equation (24) allows for the calculation of reasonable values of the activation energy. Assuming a random distribution of alkali cations, the ionic hopping distance, *a*_o_, approximated by the average cation-cation distance, is given by the following:

*a*_o_ = *N*_i_^−1/3^ (26)

where *N*_i_ is the number of alkali cations per cm^−3^. For the borate glass 0.68B_2_O_3_-0.32Li_2_O glasses, *E*_a_ = 0.82 eV and *N*_i_ = 1.5 × 10^22^ cm^−3^.

#### 6.2.4. LiPON

**Figure 23:**
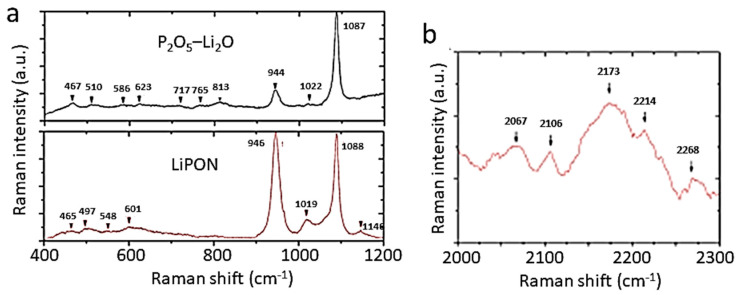
(**a**) Raman spectra of P_2_O_5_-Li_2_O and LiPON thin films deposited on a Cr layer. Spectra were recorded using a 266 nm-UV laser source. (**b**) Raman spectrum of the 1.4 µm-thick LiPON layer in the range 2000–2300 cm^−1^. Reproduced with permission from [161]. Copyright 2010 Elsevier.

Since its discovery by Bates et al. [158] in 1992, lithium phosphorus oxynitride LiPO_x_N_y_ (LiPON), an amorphous FIC, is one of the most prominent electrolytes for application in all solid-state thin-film lithium-ion batteries. The FIC film Li_3.3_PO_3.9_N_0.17_ prepared by sputtering Li_3_PO_4_ in N_2_ atmosphere has a conductivity at 25 °C of 2 × 10^−6^ S cm^−1^ and is stable in contact with Li metal [159,160]. Pichonat et al. prepared LiPON by radio-frequency sputtering of a mixture target of P_2_O_5_-Li_2_O (0.25:0.75) and studied the film structure via Raman spectroscopy [161]. The structure of phosphorus oxynitride glasses was first investigated by Bunker et al. [162]. Raman spectra of model phosphazene compounds with different types of P-N bonding have been used to confirm spectral assignments. Spectral patterns suggest that both P−N=P and P−N${<}_{P}^{P}$ bonding configurations are present in the nitrided NaPO_3_ framework. Therefore, the bands near 800 cm^−1^ in both model compounds and nitrided phosphate glass are tentatively assigned to P−N=P, while those near 600 cm^−1^ are assigned to P−N${<}_{P}^{P}$. Figure 23a presents the Raman spectrum of LiPON film deposited onto a 200 nm-thick Cr-Pt blocking electrode compared with the pattern of a P_2_O_5_-Li_2_O film. Peak attributions for both materials are summarized in Table 4. Nitrogen incorporation could be quantified to some extent through the high-frequency Raman spectrum (Figure 23b), where peaks at 2067, 2106, 2173, 2214, and 2268 cm^−1^ are due to the vibration of P−N${<}_{P}^{P}$ and P−N=P bonds.

#### 6.2.5. Garnet-Type FICs

**Figure 24:**
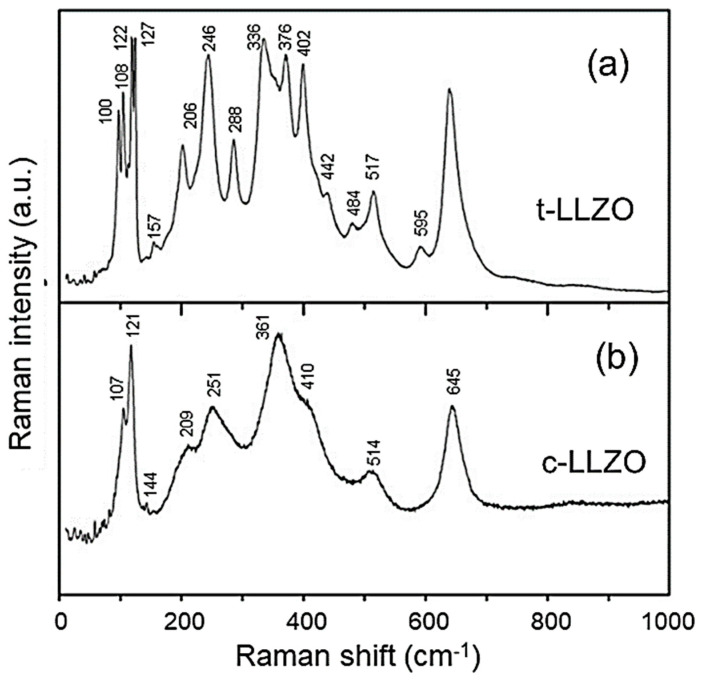
Unpolarized micro-Raman spectra of polycrystalline pellets of the LLZO solid electrolyte. (**a**) t-LLZO, after calcination at 1100 °C in a Pt crucible. (**b**) c-LLZO, sintered at 1200 °C in an Al_2_O_3_ crucible. Spectra were obtained in backscattering geometry. Reproduced with permission from [41]. Copyright 2013 Elsevier.

The Li^+^ ion conductor Li_7_La_3_Zr_2_O_12_ (LLZO) prepared by solid-state reaction after calcination at 1100 °C is a ceramic with a tetragonal structure (t-LLZO, space group *I*41/*acd*), which transforms to a cubic crystal structure (c-LLZO, space group *Ia*$\bar{3}$*d*) at 1200 °C. The LLZ tetragonal phase exhibits an ordered structure with lithium occupying the tetrahedral 8*a* site and octahedral 16*f* and 32*g* sites. In contrast, the cubic phase LLZO is disordered at the tetrahedral 24*d* Li(1) site and octahedral 96*h* Li(2) sites. As illustrated in Figure 24, the Raman spectrum of the tetragonal phase t-LLZO shows a greater number of spectral features (peaks or bands) than that of the cubic phase c-LLZO, which is due either to a more distorted arrangement of Li^+^ ions or to the weaker symmetry of the garnet-related tetragonal structure [163]. Preliminary studies of the Raman spectra of LLZO carried out by Tietz et al. highlighted that the band near 650 cm^−1^ corresponds to the elongation of the ZrO_6_ octahedra [41]. In the case of cubic garnet (*Ia*$\bar{3}$*d* space group), group theory predicts the existence of 25 active Raman modes, namely 3*A*_1g_ + 8*E*_g_ + 14*T*_2g_ [164]. During measurements performed with a standard 90° scattering geometry, the *A*_1g_ and *E*_g_ modes are polarized parallel to the laser polarization *X*(*YY*)*Z* (Porto notation), while the *T*_2g_ modes are polarized with a cross-polarization or *X*(*YX*)*Z*.

Cubic LLZO can be stabilized by doping supervalent cations on the Zr site with only 0.25 moles of Li vacancies per LLZO formula unit. Using Raman spectroscopy, Thompson et al. [165] determined the concentration of supervalent cations on the Zr required to stabilize cubic LLZO, from a critically doped composition of Li_6.5_La_3_Zr_1.5_Ta_0.5_O_12_ and a sub-critically doped composition of Li_6.75_La_3_Zr_1.5_Ta_0.25_O_12_, which crystallizes as a mixture of tetragonal and cubic phases. This was mainly evidenced by the measurements of the Raman bands near ~110 cm^−1^. The Raman fingerprints of the cubic garnet-type Li_6_La_3_Ta_1.5_Y_0.5_O_12_ include the low-frequency lattice modes between 100 and 150 cm^−1^ corresponding to vibration of the heavy La^3+^ cation and Li-ion vibrations between 300 and 600 cm^−1^. More specifically, the internal modes of octahedrally coordinated Li occur at 200–300 cm^−1^, and the modes of tetrahedrally coordinated lithium occur at 350–600 cm^−1^ [166,167]. Note that the considerable mixing between internal modes of LiO_4_, LiO_6_, and the other coordinated groups present in the structure leads to complications in the interpretation of the Raman spectra of lithium garnets. The strong peak at ~750 cm^−1^ is assigned to the stretching modes of TaO_6_ octahedra [165]. Janani et al. [166] studied the influence additive on the structure of Al-doped Li_7_La_3_Zr_2_O_12_ lithium garnet via XRD and Raman spectroscopy and established that the Al-LLZO samples sintered at 900 and 1200 °C stabilized in the highly conductive cubic phase. Raman spectrum of LLZO brown precursor sintered at 700 °C also indicated the formation of pyrochlore La_2_Zr_2_O_7_ phase. Narayanan and co-workers investigated the volatility of lithium during the preparation of lithium-stuffed garnet-type Li_6_La_3_Ta_1.5_Y_0.5_O_12_ solid Li-ion electrolytes, which is a common problem affecting phase formation, ionic conductivity, mechanical strength, and density [168]. Raman spectroscopy performed with a 532 nm laser-line shows some amount of Li_2_CO_3_ formation at the sample surface, as evidenced by the strong peak at 1090 cm^−1^. A series of Al- and Nb-co-doped Li_7−y−3x_Al_x_La_3_Zr_2−y_Nb_y_O_12_ (*y* = 0.1–0.25, *x* = 0–0.25) solid electrolytes were synthesized by the sol–gel method and sintered at 1150 °C for 1 h [169]. The phase analysis of the cubic structure performed by Raman spectroscopy corroborated the ionic conductivity associated with the amount of the charge carriers Li^+^. The highest total conductivity of 6.3·× 10^−4^ S cm^−1^ at 25 °C for Li_6.6_Al_0.05_La_3_Zr_1.75_Nb_0.25_O_12_ is viewed by the profile of Raman bands attributed to the Li-O bonds. No signature of LCO or Ta-LLZO reaction byproducts was observed in the XRD patterns and Raman spectra of the composites annealed at 1050 °C for 1 h in air [170]. The LaCoO_3_ or Co_3_O_4_ phases were not detected in the high-resolution Raman spectra. However, a weak band at 689 cm^−1^ was recorded in the spectrum of the Ta-LLZO grains, indicating that the Co concentration diffused into the Ta-LLZO grains was low.

#### 6.2.6. Nasicon-like FICs

**Figure 25:**
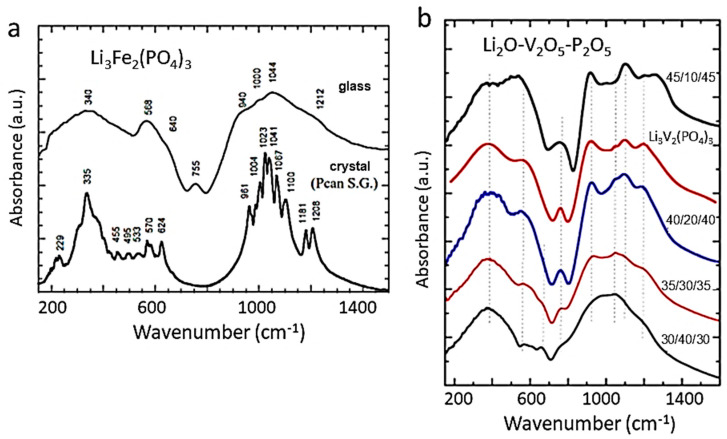
(**a**) FTIR absorption spectra of lithium iron phosphates with Nasicon-like framework for crystalline and vitreous Li_3_Fe_2_(PO_4_)_3_. Reproduced from [65]. Copyright 2006 Elsevier. (**b**) FTIR absorption spectrum of vitreous Li_5_V_2_(PO_4_)_5_ with Nasicon-like random framework compared with spectra of selected glasses of the Li_2_O-V_2_O_5_-P_2_O_5_ system. Numbers (e.g., 45/10/45) mean mol% of glass constituents. Dashed lines point out the position of the active IR modes.

The Nasicon-type LiTi_2_(PO_4_)_3_ crystal structure (LTP, space group *R*$\bar{3}$*c*) consists of a three-dimensional lattice of TiO_6_ octahedra and PO_4_ tetrahedra linked at their vertices [170,171]. In this structure, Li^+^ ions occupy interstitial sites, denoted as Li(1). Both Li and Ti atoms exhibit octahedral coordination, and the PO_4_ tetrahedron is composed of two types of oxygen atoms. One of these (O1) is also bound to a Ti^4+^ ion, while the second (O2) is bonded to both a Ti^4+^ ion and a Li^+^ ion. Nasicon-type LTP compounds have been studied by infrared and Raman spectroscopy in various forms: polycrystalline [172,173,174,175,176,177,178,179], glassy [65,180], doped [181,182,183], and single crystal [184]. Ait-Salah et al. [65] studied the local cationic arrangement of Nasicon-like glassy structures using a molecular vibration model to determine the strong covalent bonds within a PO_4_^3−^ complex. Figure 25a shows the FTIR absorption spectra of the crystalline and glassy Nasicon Li_3_Fe_2_(PO_4_)_3_ phases. The glassy spectrum corresponds approximately to the envelope of the spectrum of crystalline phosphate. In the phosphate spectra, the internal modes involving the displacement of oxygen atoms of the pseudotetrahedral PO_4_^3−^ anions exhibit frequencies very close to those of the free molecule. The splitting of the high-frequency infrared band, with components pointed at 1004, 1023, 1041, and 1067 cm^−1^, is attributed to a manifestation of a correlation field effect that originates from the coupling of the PO_4_ vibrations in the Nasicon lattice. Lantern units present in the Nasicon structure give rise to infrared bands in the 1150–1250 cm^−1^ range. These bands, attributed to the stretching modes of the terminal PO_3_ units, are located at 1181 and 1208 cm^−1^ for Li_3_Fe_2_(PO_4_)_3_. The two high-frequency spectral features and the intense band in the low-wavenumber region at 300–350 cm^−1^ are characteristic of Nasicon-type frameworks. The FTIR spectrum of Li_3_Fe_2_(PO_4_)_3_ glass sample displays an additional band centered at 755 cm^−1^, attributed to the P-O-P bond present in the disordered Nasicon phase. Figure 25b presents the FTIR absorption spectra of the [*M*_2_(PO_4_)]. The spectrum of the Li_5_V_2_(PO_4_)_5_ glass compound exhibits the same characteristics as that of the Li_2_O-V_2_O_5_-P_2_O_5_ glass system. The high-frequency band around 1180 cm^−1^ due to P-O stretching, as well as the band at 750 cm^−1^ due to P-O-P bending, can be considered evidence of the presence of PO_4_^3−^ pyrophosphate species in the glasses. A similar band was observed in Raman spectra of silver-phosphate glasses containing orthophosphate and pyrophosphate group [185], and as well as in Raman spectra of silver-vanadate glasses containing metavanadate chains [186]. Although the spectra are dominated by phosphate ion vibrations, transition-metal ions are also present in the intermediate region from 400 to 700 cm^−1^.

Barba and Frech investigated the vibrational spectra of LiTi_2_(PO_4_)_3_ [182]. The band at 487 cm^−1^ observed in the IR spectrum is attributed to the “cage vibration” mode of the Li^+^ ions due to their occupancy in the M(3) and M′(3) sites in the Li_3_Ti_2_(PO_4_)_3_ structure. A slight band broadening is also observed in the vibrational spectra during the insertion of the Li^+^ ions, which could indicate some disorder. Polarized Raman spectroscopy of a microcrystal Nasicon-type LiTi_2_(PO_4_)_3_ was investigated in backscattering geometry [187]. Raman modes have been unambiguously identified by determining both their wavenumber and symmetry. The corresponding spectra reveal that 13 different Raman modes peaked at about 92, 139, 185, 196, 240, 274, 353, 446, 457, 546, 969, 1007, and 1071 cm^−1^ are identified as an *E*_g_ mode, and seven distinct Raman modes peaked at 178, 312, 432, 450, 610, 1018, and 1095 cm^−1^ have the *A*_1g_ symmetry. Jimenez and co-workers investigated the local structure of Li_1.3_Al_0.3_Ti_1.7_(PO_4_)_3_ (LATP) thick films prepared by tape casting [188]. The confocal micro-Raman spectroscopy detected phases corresponding to LATP crystallites and rounded particles. This secondary glassy phase displayed an important band near 750 cm^−1^ attributed to lithium titanate. There was also evidence of rutile TiO_2_ domains.

#### 6.2.7. Perovskite-Type FICs

Many efforts have been invested to elucidate the crystal structure and conduction mechanism of perovskite fast-ionic conducting oxides. The compounds Li_3x_La_(2/3)−x(1/3)−2x_TiO_3_ (LLTO) exhibit a perovskite (*AB*O_3_)-type structure. The *A* sites consist of La ions, alkali-metal ions (Li^+^, Na^+^, K^+^), or rare-earth ions, located at the vertices of a cube, while the *B* sites consist of transition-metal Ti ions, located at the center of the cube. Oxygen atoms occupy the face-centered positions. Typically, the *A* and *B* sites have 12 and 6 coordination numbers (*B*O_6_), respectively, thus sharing vertices [189,190]. The La_2/3−x_Li_3x_TiO_3_ system has been extensively studied for use in Li batteries as electrodes, solid electrolytes, and as separators in lithium-air batteries as a result of its high ion mobility, which can reach up to 10^−3^ S cm^−1^ at room temperature [191,192,193]. Raman spectra were performed in order to obtain further information regarding the short-range structural organization of the LLTO samples [193,194,195,196]. The spectra present seven bands located at 140, 230, 320, 360, 400, 530, and 660 cm^−1^. The bands around 140, 230, and 530 cm^−1^ are attributed to the *E*_g_ modes, while the *A*_1g_ mode is observed at 320 cm^−1^. The modes observed for the La_(2−x)/3_Li_x_TiO_3_ crystals and their assignment are presented in Table 5. According to Sanjuán et al. [197], the bands near 140 and 320 cm^−1^ are mainly due to titanium vibrations in the plane and along the *c*-axis, respectively, while the other modes are attributed to oxygen motion. The bands located around 360, 400, and 660 cm^−1^ present in the Raman spectra of lithium titanate reveal the presence of Li_2_TiO_3_ as a secondary phase. Mei et al. [198] reported that the Raman spectrum of the pure LLTO consists of six bands at about 140, 233, 317, 457, 522, and 575 cm^−1^, which are in agreement with the prediction for a tetragonal (*P*4/*mmm* S.G.) double-perovskite cell exhibiting six Raman-active modes (2*A*_1g_ + *B*_1g_ + 3*E*_g_) involving Ti and O atoms.

### 6.3. Solid Electrolyte Interphase (SEI)

The SEI is arguably one of the most critical components of the Li-ion cell. The composition, structure, and formation mechanism of the SEI are still debated despite thousands of studies [199]. For an infrared and Raman database of battery interphase components, see Ref. [200]. Surface-enhanced Raman spectroscopy (SERS) has been claimed to be employed for in situ studies of SEI formation [201,202,203] due to the high intrinsic surface sensitivity of this technique. Schmitz et al. [204] investigated, by Raman spectroscopy, the composition and formation of the SEI formed on lithium electrochemically deposited on a Cu substrate. Semicarbonates, Li_2_CO_3_, and Li_2_C_2_ are identified as SEI components. The existence of Li_2_C_2_ is identified by a signal at 1856 cm^−1^ assigned to the symmetric stretching vibration of the triple bond of the acetylide anion. Ha et al. [202] introduced a novel electrode fabrication method (a-Si/roughened Cu mesh) to monitor the reversible electrochemical behavior of an a-Si thin-film electrode and newly formed alkyl carboxylates (R-COO^−^) within the SEI during repeated cycles. Operando Raman spectroscopy, coupled with online electrochemical mass spectrometry, is used to investigate the SEI formation on Au substrate in a LiClO_4_-based model electrolyte in ethylene carbonate (EC) solvent. The electrolyte itself and cell contaminants, such as O_2_, CO_2_, and H_2_O, contribute to SEI formation in stages. The effects associated with electrode/electrolyte double-layer charging, electrode adsorbate polarization (Stark effect), and SEI dissolution are highlighted. Lithium carbonate and lithium oxide are identified as main products formed at a potential of ≈2 V vs. Li^+^/Li [205].

**Figure 26:**
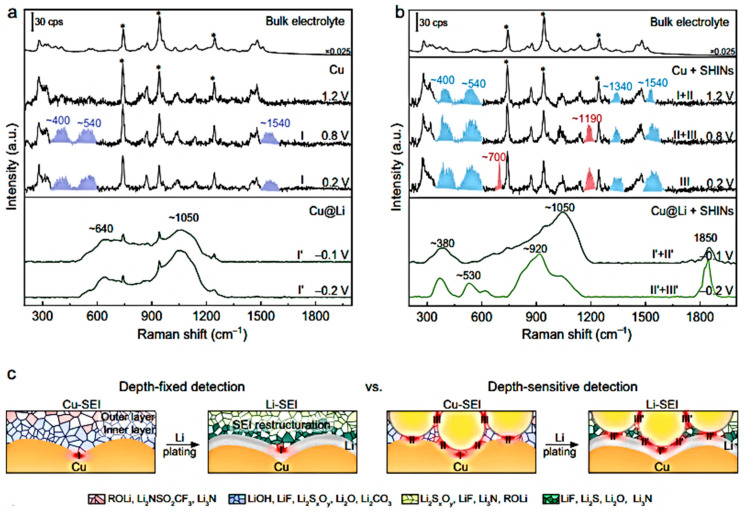
Analyses of the SEI formed in the 1 mol L^−1^ LiTFSI in DME-DOL using DS-PERS. (**a**,**b**) In situ Raman spectra of the sequential formation and evolution of SEIs on the nanostructured Cu substrate (**a**) and on the Cu-SHINs substrates (**b**) before and after Li deposition. Raman spectra of the bulk electrolyte are displayed for comparison. Peaks marked by asterisks are from the electrolyte. (**c**) Schematic comparison of the depth-fixed strategy based on LSPs of Cu alone and the depth-sensitive strategy based on synergetic LSPs of integrated Cu-SHINs for in situ probing the formation and restructuring of SEI. Reproduced from [207]. Copyright 2023 under Creative Commons Attribution CC BY 4.0 International license.

The SEI layer on anode made of silicon particles (70–130 nm) mixed with Timcal C45 carbon conductive additive and lithium polyacrylate (LiPAA) binder titrated to pH 7 in a weight ratio of 70.7:9.4:19.9 was studied using SERS [206]. Organophosphate derivatives, which are typical products of LiPF_6_ salt, were detected using a 785 nm (λ_exc_) diode laser. The vibrations observed at approximately 1217, 1244, and 1395 cm^−1^ are compatible with the stretching ν_P=O_ modes of organophosphorus compounds, such as (CH_3_)_2_P(=O)CH_3_, P(=O)F_3_, and PO_3_^2−^. Gu et al. [207] developed a depth-sensitive plasmon-enhanced Raman spectroscopy (DS-PERS) technique for the in situ and non-destructive characterization of the nanostructure and chemistry of SEI. This method relies on the synergistic amplification of localized surface plasmons from nanostructured copper nanoparticles, shell-isolated gold nanoparticles, and Li deposits at different depths. The resulting technology, called shell-isolated nanoparticle-enhanced Raman spectroscopy (SHINERS), has overcome the long-standing limitations of traditional SERS and can be employed to probe structural evolution and electrochemical reactions at the surface of non-metallic substrates. Figure 26 illustrates the in situ Raman spectra of the sequential formation and evolution of SEIs on the nanostructured copper substrate and on a shell-isolated copper nanoparticle (SHINs) substrate before and after Li deposition. Figure 26c shows a schematic comparison of the fixed-depth strategy based on localized surface plasmons (LSPs) of copper alone and the depth-sensitive strategy based on synergetic LSPs of integrated Cu-SHINs for in situ study of the formation and restructuring of the SEI. DS-PERS analysis reveals that primary Cu-SEI can restructure with the participation of freshly deposited metallic lithium to form low-oxidation-state species with highly amorphous and heterogeneous architecture.

**Figure 27:**
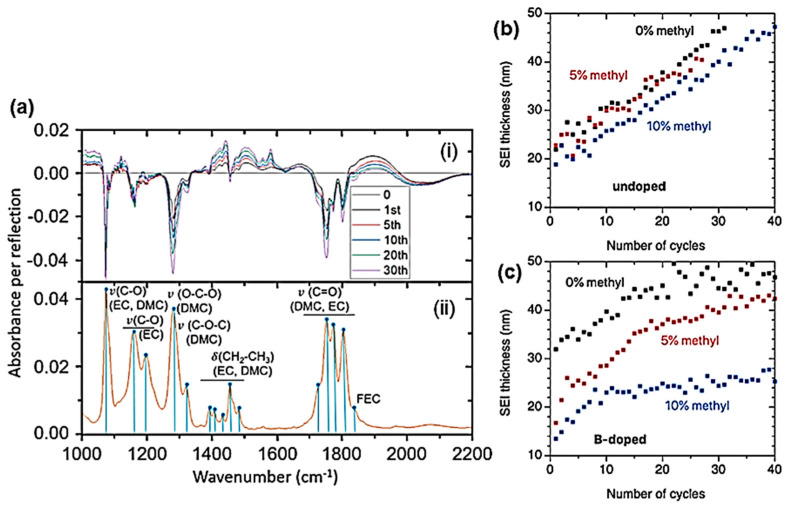
(**a**) Operando ATR-FTIR spectra of (**i**) a-Si:H electrode (30 nm thick) after successive Li insertion/extraction cycles, and (**ii**) absorbance spectrum of the electrolyte 1 mol L^−1^ LiPF_6_ in EC:DMC (1:1) with 5% FEC additive. Evolution of the SEI thickness as a function of cycle number for (**b**) a-Si:H anode and (**c**) B-doped Si. Reproduced from [208]. Copyright 2025 American Chemical Society.

The effects of methylation and boron doping on the solid electrolyte interphase (SEI) formed on amorphous Si electrodes were investigated using operando attenuated total reflectance infrared spectroscopy (ATR-FTIR) [208]. This technique enables quantitative determination of SEI thickness and provides insight into its chemical composition, including organic carbonates, lithium carbonate, and polycarbonate species. ATR-FTIR spectra are recorded in the range 1000–2200 cm^−1^ after successive 30 lithium insertion/extraction cycles (Figure 27a). The absorbance variations were evaluated as *A*(*t*) = −ln[*I*(*t*)/*I*(0)], where I(t)I(t)I(t) corresponds to the spectrum recorded at time t, and I(0) is the reference spectrum acquired at open-circuit potential (~2.8 V vs. Li^+^/Li). Quantitative analysis of SEI growth was performed by monitoring the time evolution of characteristic peak intensities. Determination of SEI thickness requires a scaling factor *k_m_*, which links the intensity of a given SEI-related vibrational mode to that of the corresponding electrolyte peak measured when the liquid electrolyte is in direct contact with the Si electrode. The SEI thickness, *d*_SEI_, can be calculated by the following:

(27) $$d_{SEI}=\frac{k_{m}d_{p}}{2},$$

where *d*_p_ is the penetration depth of the evanescent wave outside the electrode. It is given by the following:

(28) $$d_{p}=\frac{1}{2\pi \sigma {\left(n_{Si}^{2}{sin}^{2}\theta -n_{el}^{2}\right)}^{1/2}},$$

where σ is the wavenumber of the infrared radiation, *n*_Si_ and *n*_el_ are the refractive indices of the silicon, and the electrolyte at σ, respectively. In practice, the intense ν(O-C-O) vibration band of DMC at 1281 cm^−1^ was selected to estimate the scaling factor *k*_m_. At this frequency, the propagation distance *d*_p_ was calculated to be 589 nm using *n*_el_ = 1.380, *n*_Si_ = 3.418, and θ = 45°. The evolution of SEI thickness during cycling was then monitored for various electrodes. For pure amorphous Si (a-Si), the SEI growth is represented in black, whereas 5% and 10% methylated a-Si electrodes are shown in red and blue, respectively (Figure 27b). The behavior of a boron-doped sample is presented in Figure 27c, enabling direct comparison of the influence of methylation and boron doping on SEI formation and stability.

### 6.4. Cathode Electrolyte Interphase (CEI)

Similar to SEI, a film generated on the cathode surface during cycling, called the cathode electrolyte interphase (CEI) film, plays an equally important role in the performance of secondary batteries [209,210]. Studies of CEI have been mainly carried out using in situ FTIR spectroscopy [211,212,213,214,215,216] and by Raman spectroscopy (i.e., SERS and SHINERS) [11,217,218]. For instance, Meng et al. [216] reported the operando ATR-FTIR investigation of CEI dynamic reversible evolution on the Li/Mn-rich Li_1.2_Ni_0.2_Mn_0.6_O_2_ cathode in cells with carbonate-based electrolytes with or without tris(trimethylsilyl)borate (TMSB) additive. It has been shown that the components of the CEI are primarily generated during the first cycle by the preferential decomposition of ethylene carbonate (EC). Furthermore, the presence of TMSB can partially inhibit EC decomposition and alter the stability of the CEI film.

Wu et al. [219] combined in situ and ex situ FTIR characterizations to study the aging mechanism of NCA cathodes. With increasing cutoff voltage, the cathode–electrolyte interphase (CEI) at the NCA interface undergoes a three-phase evolution: formation, thickening, and then rupture. This phenomenon is closely linked to the H2-H3 phase transition. The volumetric stresses and strains induced by this transition accelerate the formation and propagation of secondary microcracks in the electrode material, thus increasing the variations in the CEI interface. Using in situ attenuated total reflectance Fourier transform infrared (ATR-FTIR) methods, Tremolet de Villers et al. [220] detected the formation and evolution of a CEI surface layer on the NMC622 cathode that contributes to the cell’s capacity fade. They monitor the CEI interactions in a coin-type cell with 1.2 mol L^−1^ LiPF_6_ in EC:EMC, 3:7 wt.%. Specifically, advanced spectral analysis elucidates changes in near-surface Li^+^ ion (de)solvation by solvent molecules during galvanostatic cycling.

The rapid development of spectroscopic techniques—particularly in situ and operando methods—continues to expand our ability to resolve dynamic structural and chemical processes with unprecedented precision. For example, in situ Raman spectroscopy has proven highly effective for monitoring structural evolution and key reaction intermediates on Cu-based catalysts during electrochemical CO_2_ reduction (CO_2_RR). Time-resolved Raman spectroscopy, combined with surface-enhanced Raman scattering (SERS), enables direct visualization of local pH fluctuations at the electrode–electrolyte interface, providing molecular-level insights into mechanisms governing reaction selectivity.

More advanced analytical strategies integrate high-resolution confocal Raman spectroscopy with complementary in situ techniques such as X-ray absorption spectroscopy and synchrotron-based FTIR. These multimodal approaches reveal multiscale correlations between catalyst structure and performance, including intrinsic relationships between CO_2_RR activity and the evolution of the Cu^0^/Cu^+^ ratio [221]. Hy et al. further demonstrated that SiO_2_-coated Au nanoparticles can serve as robust SERS probes to monitor solid electrolyte interphase (SEI) formation during electrochemical cycling in real time [201].

Recently, emerging nanoscale spectroscopies—most notably tip-enhanced Raman spectroscopy (TERS)—have opened new avenues for probing interfacial processes at the micro- and nano-meter scales. Laurenti and co-workers applied operando TERS to track chemical and structural transformations at buried interfaces during battery operation. Their work resolved nanoscale Li-ion dynamics in LiMn_2_O_4_ and LiFePO_4_ thin films, revealing a delayed appearance of the λ-MnO_2_ phase at grain boundaries during LiMn_2_O_4_ delithiation, consistent with accelerated Li^+^ diffusion in these regions. In contrast, LiFePO_4_ exhibited significantly reduced spectral visibility under similar conditions [222].

## 7. Concluding Remarks

In this chapter, Raman and FTIR spectroscopies are highly effective analytical methods in an intensive field of research covering all aspects of battery operation. They are non-destructive and relatively low-cost techniques. All battery components are involved: anodes, cathodes, liquid, and solid electrolytes. FTIR and Raman spectroscopies are powerful tools for studying the short-range order (local environment) in lattices. As vibrational properties are extremely sensitive to small changes in the local structure, both can provide valuable insight into the site occupation in battery materials and, accordingly, into the mechanism of ionic conductivity.

Regarding the structural response of electrode materials, the contribution of in situ Raman spectroelectrochemistry and Raman mapping is crucial for studying real-time charge–discharge cycling. In situ analyses allow the monitoring of several phenomena occurring during charging and discharging processes of lithium batteries, such as phase stability, structural changes in electrode materials during insertion/extraction reactions, interface evolution, material compatibility, the kinetics of Li^+^ ions in the electrodes and electrolytes, electrode degradation, and SEI layer formation. Like other in operando analytical methods, Raman spectroscopy is utilized not only to improve the cycle stability of batteries, but also to provide experimental evidence of new insights into the Li-ion insertion/extraction mechanism.

However, due to its shallow optical penetration depth, Raman spectroscopy primarily probes the sample surface. Therefore, the spectral response cannot be directly extrapolated to the bulk material, which can lead to discrepancies compared to in situ X-ray diffraction results, especially if the data acquisition rate is insufficient. Finally, far-infrared spectroscopy has also been proven effective for studying lithium-ion dynamics, providing ionic conductivity and charge carrier mobility as a function of frequency.

## Figures and Tables

**Table 1:** The cation–oxygen internuclear distance *r*_M-O_ for coordination numbers 4 ≤ *CN* ≤ 12. Reproduced from [3]. Copyright 2014 Springer.

Cation	*r_M-O_* (Å)				
	4	6	8	10	12
Li	1.99	2.16	2.32	-	-
Na	2.39	2.42	2.58	-	-
K	2.77	2.78	2.91	2.99	3.04

**Table 2:** Symmetry and Raman-active modes of the various LTMOs’ structures.

Type of Structure	Space Group	Raman Activity
Layered hexagonal rock-salt	$D_{3d}^{5}-R\bar{3}m$	*A*_1*g*_ + *E_g_*
Layered monoclinic rock-salt	$C_{2h}^{3}$–*C*2/*m*	2*A_g_ +* 2*B_g_*
Normal cubic spinel	$O_{h}^{7}$–*Fd3m*	*A*_1*g*_ *+ E_g_ +* 3*F*_2*g*_
Modified cubic spinel	$O_{h}^{7}$–*Fd3m*	*A*_1*g*_ *+ E_g_ +* 3*F*_2*g*_
Normal tetragonal spinel	$D_{4h}^{19}$–*I4*_1_/*amd*	2*A*_1*g*_ *+ B*_1*g*_ *+* 3*B*_2*g*_ *+* 4*E_g_*
Inverse cubic spinel	$O_{h}^{7}$–$Fd\bar{3}m$	*A*_1*g*_ *+ E_g_ +* 3*F*_2*g*_
Ordered cubic spinel (I)	*O^7^*–*P4*_1_*32*	6*A*_1_ *+* 14*E +* 20*F*_2_
Ordered cubic spinel (II)	$D_{4}^{3}$–*P4*_1_*22*	9*A*_1_ *+* 10*B*_1_ *+* 11*B*_2_ *+* 21*E*
Ordered cubic spinel (III)	$T_{d}^{2}$–$F\bar{4}3m$	3*A*_1_ *+* 3*E +* 6*F*_2_

**Table 3:** Characteristic frequencies (cm^−1^) of the SO_2_ and CF_3_ stretching vibrations of TFSI. Reproduced from [124]. Copyright 1989 Elsevier.

Vibration	(H_2_O)_266_/LiFTSI at 25 °C		P(EO)_15_/LiTFSI at 80 °C	
	IR	Raman	IR	Raman
*ν*_a_(SO_2_)	1354, 1333	1354, 1334	1349, 1325	1350, 1330
*ν*_s_(SO_2_)	1136	1140	1143–1135	1131
*ν*_a_(CF_3_)	1193	-	1206	-
*ν*_s_(CF_3_)	-	1243	-	1239
*ν*_a_(SNS)	1060	-	1055	-

**Table 4:** Assignment of the Raman peaks for the P_2_O_5_-Li_2_O and LiPON solid electrolyte.

Frequency (cm^−1^)		Assignment
P_2_O_5_-Li_2_O	LiPON	
467	465	Li(2)-O stretching
510, 586, 623, 944	497, 946	P-O bond in orthophosphate PO_4_^3−^
	601, 625	P−N${<}_{P}^{P}$ bond
	802	P−N=P bond
1022	1019	P-O bond in pyrophosphate P_2_O_7_^4−^
	1146	P-O bond in metaphosphate (PO^3−^)_n_

**Table 5:** Observed modes of La_(2−x)/3_Li_x_TiO_3_ crystal and attribution with their major contribution, though some admixture between displacements of the same symmetry. Reproduced from [196]. Copyright 2005 American Chemical Society.

Raman Shift (cm^−1^)	Symmetry	Displacements
140	*E*_g_	Ti in-plane
230	*E*_g_	O(3) in-plane
315	*A*_1*g*_	Ti *c*-axis
450	*A*_1*g*_ (forbidden)	O(1,2) *c*-axis
525	*E*_g_	O(3) in-plane
550, 580	*A*_1*g*_ (allowed)	O(3) *c*-axis

## Data Availability

No new data were created or analyzed in this study. Data sharing is not applicable to this article.

## Abbreviations

The following abbreviations are used in this manuscript:

3D	Three-dimensional
ASSBs	All-solid-state batteries
ATR	Attenuated total reflectance
BP	Boson peak
BWF	Breit–Wigner–Fano
CE	Counter electrode
CEI	Cathode electrolyte interphase
CN	Coordination number
DME	Dimethoxyethane
DOL	Dioxolane
EC	Ethylene carbonate
EMC	Ethyl methyl carbonate
FTIR	Fourier transform infrared
FIC	Fast ionic conductor
FWHM	Full-width-at-half-maximum
KJMA	Kolmogorov–Johnson–Mehl–Avrami
LATP	Li_1.3_Al_0.3_Ti_1.7_(PO_4_)_3_
LCO	LiCoO_2_
LFP	LiFePO_4_
LGPS	Li_10_GeP_2_S_12_
LIBs	Lithium-ion batteries
LiPON	Lithium phosphorus oxynitride
LiTFSI	LiN(SO_2_CF_3_)_2_
LLTO	Li_3x_La_(2/3)−x(1/3)−2x_TiO_3_
LLZO	Li_7_La_3_Zr_2_O_12_
LPS	Li_7_P_3_S_11_
LNM	Li[Ni_0.5_Mn_1.5_]O_4_
LNO	LiNiO_2_
LSPS	Li_10_SiP_2_S_12_
LSPSO	Li_10_SiP_2_S_12−x_O_x_
LTO	Li_4_Ti_5_O_12_
LTP	LiTi_2_(PO_4_)_3_
NBO	Non-bridging oxygen
NCA	LiNi_0.8_Co_0.15_Al_0.05_O_2_
NMC	LiNi_1−x−y_Mn_x_Co_y_O_2_
PEO	Poly(ethylene oxide)
PERS	Plasmon-enhanced Raman spectroscopy
RE	Reference electrode
RRS	Resonance Raman spectroscopy
RS	Raman scattering
TEGDME	Tetraethylene glycol dimethyl ether
TMO	Transition-metal oxide
TMSB	Tris(trimethylsilyl)borate
SEI	Solid electrolyte interphase
SERS	Surface-enhanced Raman spectroscopy
SHIN	Shell-isolated nanoparticle
SIS	Solvent-in-salt
SOC	State of charge
SSE	Solid-state electrolyte
SSR	Solid-state reaction
TERS	Tip-enhanced Raman spectroscopy
VC	Vinylene carbonate
WE	Working electrode
XRD	X-ray diffraction
